# Trichomes and unique gene expression confer insect herbivory resistance in *Vitis labrusca* grapevines

**DOI:** 10.1186/s12870-024-05260-9

**Published:** 2024-06-27

**Authors:** Cullen W. Dixon, Andrea R. Gschwend

**Affiliations:** 1 Department of Horticulture and Crop Science, The Ohio State University, Columbus, OH 43210 USA; 2 Center for Applied Plant Sciences, The Ohio State University, Columbus, OH 43210 USA

**Keywords:** *Vitis labrusca*, *Vitis vinifera*, Insect herbivory, Japanese beetle, Comparative transcriptomics, Trichomes, Terpenes, Flavonoids

## Abstract

**Background:**

Grapevine (*Vitis*) is one of the world’s most valuable fruit crops, but insect herbivory can decrease yields. Understanding insect herbivory resistance is critical to mitigating these losses. *Vitis labrusca*, a wild North American grapevine species, has been leveraged in breeding programs to generate hybrid grapevines with enhanced abiotic and biotic stress resistance, rendering it a valuable genetic resource for sustainable viticulture. This study assessed the resistance of *V. labrusca* acc. ‘GREM4’ and *Vitis vinifera* cv. ‘PN40024’ grapevines to *Popillia japonica* (Japanese beetle) herbivory and identified morphological and genetic adaptations underlying this putative resistance.

**Results:**

‘GREM4’ displayed greater resistance to beetle herbivory compared to ‘PN40024’ in both choice and no-choice herbivory assays spanning periods of 30 min to 19 h. ‘GREM4’ had significantly higher average leaf trichome densities than ‘PN40024’ and beetles preferred to feed on the side of leaves with fewer trichomes. When leaves from each species that specifically did not differ in trichome densities were fed on by beetles, significantly less leaf area was damaged in ‘GREM4’ (3.29mm^2^) compared to ‘PN40024’ (9.80mm^2^), suggesting additional factors beyond trichomes contributed to insect herbivory resistance in ‘GREM4’. Comparative transcriptomic analyses revealed ‘GREM4’ exhibited greater constitutive (0 h) expression of defense response and secondary metabolite biosynthesis genes compared to ‘PN40024’, indicative of heightened constitutive defenses. Upon herbivory, ‘GREM4’ displayed a greater number of differentially expressed genes (690) compared to ‘PN40024’ (502), suggesting a broader response. Genes up-regulated in ‘GREM4’ were enriched in terpene biosynthesis, flavonoid biosynthesis, phytohormone signaling, and disease defense-related functions, likely contributing to heighted insect herbivory defense, while genes differentially expressed in ‘PN40024’ under herbivory were enriched in xyloglucan, cell wall formation, and calcium ion binding. The majority of genes implicated in insect herbivory defense were orthologs with specific expression patterns in ‘GREM4’ and ‘PN40024’, but some paralogous and genome-specific genes also likely contributed to conferring resistance.

**Conclusions:**

Our findings suggest that ‘GREM4’ insect herbivory resistance was attributed to a combination of factors, including trichomes and unique constitutive and inducible expression of genes implicated in terpene, flavonoid, and phenylpropanoid biosynthesis, as well as pathogen defense.

**Supplementary Information:**

The online version contains supplementary material available at 10.1186/s12870-024-05260-9.

## Background

Grapes are the most valuable fruit crop globally [1]. In the United States, the wine industry alone had a $275B impact on the economy in 2022 [2]. Insect pests invoke up to 30% of crop loss each year globally, decreasing yields [3, 4]. To protect grapevines, growers implement integrated pest management (IPM) plans, which incorporate cultural, biological, mechanical, and physical controls to mitigate yield losses and decrease insect pressure [5–7]. Some examples of strategies include exclusion [8], trapping [9], planting resistant varieties [10], and biocontrol [11, 12]. However, if these methods are insufficient, chemical controls (insecticides) are often implemented, and are typically effective [13], but can have detrimental environmental effects [14, 15].

*Popillia japonica* (Japanese beetle) is a major polyphagous invasive pest in North America and Europe, damaging plants of both commercial and non-commercial uses, including grapevine [16–23]. While Japanese beetle grubs primarily feed on the roots of grass and other plants in the spring, once they pupate in the early to mid-summer, adults emerge from the soil and feed on above-ground portions of plants, such as grapevine, for the remainder of the summer [19, 24, 25]*.* The adult developmental stage of Japanese beetles overlaps with the vegetative, flowering, and fruit set stages of grapevine (June–August), so beetle herbivory during this time can be detrimental, as it reduces photosynthetic capacity and sugar production, affecting end-of-season fruit quality [25, 26]. Though Japanese beetle infestations can be chemically controlled with weekly or biweekly spraying regimes starting when ~ 15% of the leaf area is damaged [24], improving innate resistance of cultivated grapevines to Japanese beetles, and other insect pests, proves a promising option for growers to decrease inputs, costs, and insecticide use, while increasing yields in this multi-billion-dollar industry.

*Vitis labrusca* is a grapevine native to North America and is highly fit in its local environment. *Vitis labrusca* is cold-hardy [27, 28] and resistant to pathogens [29–33]. Conversely, *Vitis vinifera*, a species cultivated across the globe and well adapted to European biomes, is highly susceptible to abiotic and biotic stresses endemic to North America [24, 28, 34–37]. *Vitis labrusca* has been widely employed in grapevine breeding programs to introduce these adaptive traits into hybrids [34, 36]. Intriguingly, grapevine varieties bred from North American species experienced decreased insect herbivory in the field; hybrid grapevines with a majority of *V. labrusca* genetic background exhibited greater resistance to Japanese beetle, whereas *V. vinifera* cultivars and hybrids with little *V. labrusca* genetic background exhibited greater damage [23]. Further, hybrids bred from other North American grapevine species also exhibit decreased Japanese beetle herbivory and decreased mealybug (*Planococcus ficus*) infestation compared to European grapevines [21, 38]. These results suggest the genetic composition of *V. labrusca* provides an advantage for insect herbivory resistance.

Insect herbivory defense has been well studied in many plant species, though plant responses, and their efficacy, can differ depending on the pest and the plant. Perception of herbivory is the initial step in the plant immune response which initiates downstream inducible responses to the stress and, subsequently, defense [39–43]. This response is initiated by molecules known as pathogen-associated molecular patterns (PAMPs), herbivory-associated molecular patterns (HAMPs), or damage-associated molecular patterns (DAMPs). These molecules are detected by cellular receptors which initiate defense responses via signaling cascades, such as the mitogen activated phosphorylation signaling pathway (MAPK signaling) [42, 44, 45]. One of the many outcomes of such signaling pathways is increased production of secondary metabolites. The types and quantities of secondary metabolites produced in defense of insect herbivory can vary greatly between plants, but terpenes are one such secondary metabolite with known insecticidal properties. For example, essential oils containing terpenes derived of Cassumunar ginger (AKA – Plai) (*Zingiber cassumunar*) displayed insect repellent and larvicidal properties against Asiatic tiger mosquito (*Aedes albopictus*) [46]. In rice, 25 Terpene synthase (*TPS*) genes, which are critical in catalyzing terpene synthesis, were differentially expressed upon Asiatic rice borer (*Chilo suppressalis*) herbivory and overexpressing a *TPS* gene (Beta-ocimene synthase (*OCS*)) in both tobacco and soybean resulted in enhanced resistance to tobacco cutworm (*Spodoptera litura*) [47, 48]. Additionally, other secondary metabolites, such as flavonoids, play important roles in insect herbivory defense and resistance such as observed in wheat, rice, tea, sorghum, and maize [49–53]. For example, in resistant cassava (*Manihot esculenta*), increased accumulations of phenylpropanoid and flavonoid pathway compounds were identified upon two-spotted spider mite (*Tetranychus urticae*) herbivory and led to greater resistance when genes associated with their biosynthesis were overexpressed [54]. While terpenes, flavonoids, and other secondary metabolites are critical to insect herbivory defense, physical adaptations, such as trichomes, hair-like structures on the surface of plant tissues, also provide increased defense against pathogens and insect pests [36, 55–57]. High trichome densities have led to decreased insect herbivory in wheat, *Datura stramonium,* and soybean, among other plants [56, 58, 59]. These observations suggest specialized physical and metabolic defenses have evolved in resistant plants.

Limited studies have been conducted in grapevine to identify the unique adaptive defenses involved in deterring insect herbivory in North American wild grapevine species. A comparative genomic study between *V. labrusca*, *V. riparia*, and *V. vinifera* varieties identified genome-specific genetic variation linked to adaptive traits, laying the foundation for discovering the genetics that underlie adaptive differences [60]. In an herbivory study, oriental longheaded grasshopper feeding on *V. vinifera* x *V. labrusca* hybrid ‘Kyoho’ induced transcriptomic, phytohormonal, and metabolomic alterations after 72 h of feeding, with increased expression of genes implicated in reactive oxidative species (ROS) production, flavonoid biosynthesis, insect and physical damage response, and lignin biosynthesis, among others [61]. In a *V. riparia* hybrid, a quantitative trait loci (QTL) associated with phylloxera resistance was found to contain disease resistance genes, such as Resistance to Phytophthora sojae 5 (*Rps5*), which suggests genes canonically associated with pathogen resistance may also impact insect herbivory defense [57]. A comparative transcriptomic study between *V. labrusca* and *V. vinifera* in response to insect herbivory is still needed to identify specific defensive processes that could potentially contribute to insect herbivory resistance in *V. labrusca*.

In this manuscript, we conducted a comprehensive, comparative study to determine the morphological and transcriptomic factors contributing to insect herbivory defense in *V. labrusca* acc. ‘GREM4’ (‘GREM4’) and *V. vinifera* cv. ‘PN40024’ (‘PN40024’). Since *V. labrusca* was reported to exhibit heightened resistance to pathogens and pests, as discussed above, we hypothesized that ‘GREM4’ would demonstrate enhanced insect herbivory resistance compared to 'PN40024'. Further, we hypothesized that this resistance would be partly conferred by increased trichome density on leaves, along with increased expression of secondary metabolite biosynthesis genes in ‘GREM4’ compared to ‘PN40024’. To test our hypotheses, we performed Japanese beetle feeding assays to test ‘GREM4’ and ‘PN40024’ for insect herbivory resistance and determine the role of trichome density in deterring insect herbivory. Additionally, we conducted a quantitative comparative transcriptomic study to determine transcriptomic responses, and functional implications, for each species (‘GREM4’ vs. ‘PN40024’) in response to Japanese beetle herbivory and identified specific responses in ‘GREM4’ that likely contribute to insect herbivory resistance.

## Methods

### Plant materials

*Vitis labrusca* acc. ‘GREM4’ (PI-588583) and *Vitis vinifera* cv. ‘PN40024’ (DVIT-908) grapevine cuttings were acquired from the United States Department of Agriculture at Geneva, NY and Davis, CA, respectively, in 2019, 2021, and 2022, for the experiments conducted those years [62, 63]. ‘PN40024’ was selected due to its role as the *V. vinifera* reference cultivar/reference genome since 2007, while ‘GREM4’ was selected due to the availability of a reference genome sequence and its resistance to pathogens, suggesting broad fitness in its local environment [30, 60, 64]. Both species were propagated from cuttings and grown in the Howlett greenhouses at The Ohio State University, Columbus OH, USA under 16 h light:8 h dark (elevation = 228 m, latitude = 40.00212, longitude = -83.02838). All experiments took place between the months of July and October with rooted vegetative grapevines.

### Insect collections

Adult *Popillia japonica* (Japanese beetles) of similar size were collected from The Ohio State Waterman Agricultural and Natural Resources Laboratory, Columbus OH, USA between the months of July and October of 2021 and 2022 and were used for experiments within one day of collection. Beetles were collected using “Spectracide Bag-A-Bug Japanese Beetle Trap2” pheromone traps in a soybean field which had not been sprayed with insecticides. Beetles were kept in a 16.5 × 16.5 x 30in ‘bug dorm’ within a growth chamber overnight and semi-starved (one small *V. vinifera* leaf provided to prevent death due to starvation or dehydration) and were used for experiments the following day. The growth chamber was set to a 16 h light:8 h dark cycle at 25°C and 21°C, respectively. Only beetles which were actively moving in the bug dorm were used to ensure vigorous individuals were selected for the experiments.

### Herbivory preference study

Fifteen semi-starved Japanese beetles were placed in a bug dorm inside a growth chamber as previously described. Three mature, similar-sized, attached ‘GREM4’ and ‘PN40024’ leaves were concurrently introduced into the bug dorm from one-year-old vegetative plants. The experiment permitted 19 h of *ad libitum* feeding (6PM-1PM the following day) and was replicated four times between August and September of 2021. Pictures of the leaves were taken before and immediately after the allotted feeding time and total leaf areas were measured using ImageJ, with the difference in mm^2^ representing the area of feeding (AOF), i.e. the area in mm^2^ eaten by Japanese beetles [65]. Holes made completely through the leaf and noticeable tissue loss along the leaf margin were included in the AOF calculation. Significance was determined using MiniTab21 via a one-sided two-sample t-test (variances unequal) [66].

### Herbivory time course study

One semi-starved Japanese beetle was placed in a transparent, mesh 11 cm x 10 cm bag, which was then placed over one mature attached leaf of either ‘GREM4’ or ‘PN40024’ two to three-year-old vegetative plants, and beetles were permitted to feed for 30 min, 1 h, or 4 h (Supplementary Material 1: Additional Fig. 1A). 30 min was chosen since transcriptomic differences *in planta* have been observed within 20 min after encountering a stress [67]. 4 h was chosen since defensive compounds were found to increase consistently up to 4 h in a previous insect herbivory study [68]. Feeding timing began once visible damage to the leaf was observed. All experimental ‘runs’ (an attempt at collecting feeding data by placing a beetle in a bag on a leaf) were conducted in the Howlett greenhouse August through September of 2021 and 2022, between 9:00AM and 3:00PM daily. Plants were not used again for at least four days between runs to ensure *in planta* responses captured were not a vestige of prior feeding. If a beetle did not feed within a 4 h timeframe the run was considered ‘unsuccessful’. Additional runs were needed for some time points to attain the desired experimental replicates, thus ‘GREM4’ had more experimental attempts, since many ‘GREM4’ runs were unsuccessful (scored as an AOF of zero). Replicates for each condition are as follows: ﻿‘GREM4﻿’﻿ 30 min = 19; ‘GREM4﻿’ 1 h = 20; ‘GREM4﻿’ 4 h = 20; ‘PN40024﻿’ 30 min = 8; ‘PN40024﻿’ 1 h = 9; ‘PN40024﻿’ 4 h = 9.

After each run, leaves were immediately photographed, placed inside 50 mL conical tubes, then plunged into liquid nitrogen. Leaves were stored at -80°C until RNA isolation for RNA-sequencing (see “RNA Isolation and Sequencing”). ‘0 h’ control leaves were also collected, but from a different plant than the herbivory samples to avoid confounding transcriptomic responses due to the removal of a leaf.

AOF measurements were ascertained as previously described (see “Herbivory Preference Study”). The differences between ﻿‘GREM4﻿’ and ﻿‘PN40024﻿’ AOF for each herbivory time point was determined via a two-sample t-test (unequal variances) using MiniTab21 [66]. The feeding success rate for ‘GREM4’ and ‘PN40024’ was also reported as the percentage of successful feeding runs out of the total number of runs.

### Leaf trichome density observations

Trichome densities were recorded for the adaxial and abaxial sides of three immature and three mature ‘GREM4’ and ‘PN40024’ leaves from two to three-year-old vegetative plants, all plants being grown in the greenhouse, three measurements each, 72 in total. Monochrome images were obtained using a digital Nikon Eclipse 80*i* microscope at 10 × magnification with a Nikon DS_QiMc Digital Sight camera at the Molecular and Cellular Imaging Center — South, The Ohio State University. Images were scored by three independent scorers based on the *Organisation Internacionale Vitis de la Vigne et du Vin* (OIV) ‘Mature leaf: density of prostrate hairs between main veins on lower side of blade’ scale, where a score of ‘1’ indicated no trichomes were present, while ‘9’ was extremely high trichome density [69]. All trichomes, both prostrate (AKA – ‘ribbon’) and simple, were included in scoring. Significance was determined via a one-way ANOVA (Games-Howell with grouping, equal variances, confidence level 95%, error rate 0.05%) using MiniTab21 [66].

### Herbivory under equal trichome densities study

The adaxial sides of mature ‘GREM4’ and ‘PN40024’ leaves were found to not significantly differ in trichome densities (see “Results”). As such, one beetle was restricted to the adaxial side of a mature leaf for both species via a transparent plastic container with small holes for air movement and allowed to feed for 1 h on six-month-old vegetative plants (Supplementary Material 1: Additional Figs. 1B and 1C). Experiments were performed in the Howlett greenhouse between August and September of 2022. Photos of the leaves were taken before and after feeding and used to calculate the AOF as previously described (see “Herbivory Preference Study”). Runs where beetles forced their way onto the abaxial side and fed were excluded. Ten replicates were collected per species. Significance was determined via a one-sided two-sample t-test (variances equal), using MiniTab21 [66].

### ‘GREM4’ Herbivory under differing trichome densities study

Experimental conditions were identical to the “Herbivory Under Equal Trichome Densities Study”, but Japanese beetles were presented with the adaxial or abaxial sides of mature ‘GREM4’ leaves, which significantly differed in trichome density (see “Results”). A total of 10 abaxial and 21 adaxial replicates were performed in July 2023. Differing numbers of replicates were due to beetles occasionally forcing their way to the non-presented side of the leaf, resulting in greater adaxial feeding datapoints. Significance was determined as previously described (see “Herbivory Under Equal Trichome Densities Study”).

### RNA isolation and sequencing

RNA was isolated from the 30 min, 1 h, and 4 h ‘Herbivory’ and 0 h ‘Control’ leaf samples collected during the 2021 “Herbivory Time Course Study”. RNA from leaf samples was isolated using a Sigma-Aldrich Spectrum Plant Total RNA Kit and RNA quality and quantity were determined via Nanodrop, Qubit, and a formaldehyde gel. A total of 32 samples (four herbivory replicates for each of the three time points, plus four 0 h samples, for both species) were submitted to Novogene for individual library preparation and Illumina NovaSeq 6000 paired-end RNA sequencing (150 bp, 20 M reads per sample). RNA-seq reads were subjected to quality control assessments via FastQC and removal of adapters and poor quality reads via Trimmomatic [70, 71].

### *Vitis labrusca* acc. ‘GREM4’ gene annotation generation

To ensure a high quality gene annotation for downstream transcriptomic analysis, the *Vitis labrusca* acc. ‘GREM4’ genome annotation [60] was updated using ‘GREM4’ RNA-seq data to improve annotation accuracy and can be found on GitHub at https://github.com/cdixo/Vitis-labrusca-Version-2-Genome-Annotation.git as Version 2. Gene annotation was completed using the repeat masked ‘GREM4’ primary genome sequence assembly and employing Funannotate assisted by a publicly available container [60, 72–78]. BUSCO was run on the 37,443 annotated genes and 96.7% of the 1,375 BUSCO genes were detected, suggesting a high-quality annotation. Additional information can be found in Additional Fig. 2 (Supplementary Material 1) and on GitHub at https://github.com/cdixo/Inter-species-and-Herbivory-Publication.git.

### Orthologous, paralogous, and genome-specific gene identification

Orthologous genes were identified between ‘GREM4’ and ‘PN40024’ using OrthoFinder V2.2.5, DIAMOND, and custom scripts [79, 80]. Additional information can be found in Additional Fig. 3 (Supplementary Material 1) and on GitHub at https://github.com/cdixo/Inter-species-and-Herbivory-Publication.git. A subset of orthologous genes were manually checked and verified for accuracy using NCBI BLAST [81].

Genes which did not have an orthologous gene identified between the two genomes were characterized as either paralogous or genome-specific. Paralogous genes did not have a direct corresponding ortholog in the other species but did share sequence similarity to other gene(s) within the same species (i.e. were grouped into the same orthogroup by OrthoFinder). Genome-specific genes did not have a corresponding ortholog in the other species nor a paralog within the same species.

When investigating gene families which differed in size and the additional gene family members were significantly differentially expressed upon beetle herbivory, gene families were defined as orthogroups reported via OrthoFinder [79]. If multiple genes with different names were clustered into one orthogroup, the gene name present most frequently was used to name the group.

### RNA-seq read alignment, transcriptomic analysis, and enrichment analysis

RNA-seq reads were aligned to their respective genomes using STAR [82]. CoCo via ‘coco correct_counts’ was used to create the count matrix (to better account for multi-mapping reads) [83]. DESeq2 was used to identify differentially expressed genes (DEGs) [84]. DEGs were identified at each time point (30 min, 1 h, and 4 h) independently and then combined for downstream analysis. Throughout analyses, significant *p*- and *p*-adj values were defined as ≤ 0.05 whereas |log2FoldChange| was ≥ 2. RNA-sequencing reads are available at the NCBI BioProject PRJNA1070606 and RNA-seq read quality statistics are found in Additional Table 1 (Supplementary Material 2). Additional information on the pipeline and programs used to analyze the RNA-seq data can be found in Additional Fig. 4 (Supplementary Material 1) and on GitHub at https://github.com/cdixo/Inter-species-and-Herbivory-Publication.git. BioVenn, BioInfoRx, and molbiotools were used to identify DEGs conserved between transcriptomic comparisons and to create Venn diagrams [85–87].

Inter-species transcriptomic comparisons were conducted by three different methods (Supplementary Material 1: Additional Fig. 5). The first method simply determined if DEGs identified between insect herbivory samples compared to 0 h in one species were also independently determined to be DEGs in the other species. This analysis was conducted by reviewing the names of the DEGs identified, for any herbivory time point, between the two species, to determine if the DEG (gene name) was present in both lists by running an intersection command. This method was called ‘Overlap Analysis’. The second method was an ‘Interaction Analysis’ which identified genes that, upon evaluating the interaction between the genotype (‘GREM4’ or ‘PN40024’) and the treatment (herbivory or 0 h), were determined to be significantly differentially expressed. Functionally, this method explored the change in log2FoldChange (Δlog2FoldChange) between ‘GREM4’ and ‘PN40024’ for a gene, i.e. identified genes with significantly different responsiveness to insect herbivory between the two species. Technically, first, log2FoldChange values were generated for the 30 min, 1 h, or 4 h herbivory samples, compared to 0 h, for all orthologous genes in their respective species. Then, these log2FoldChange values were compared between species to identify log2FoldChange values that were significantly different (|Δlog2FoldChange|≥ 2; *p*-adj ≤ 0.05). A third method to compare inter-species expression was required, since a significantly different Δlog2FoldChange could be reported for a gene between the two species without the gene ultimately being differentially expressed between the two species (Supplementary Material 1: Additional Fig. 5). For this reason, ‘Cross-Reference Analysis’ was also conducted. Functionally, this method identified which genes had significantly different expression at a time point between species. Technically, this analysis first identified DEGs from herbivory for 30 min, 1 h, or 4 h time points compared to 0 h for all orthologous genes (via DESeq2), in their respective species, and then compared expression (read count values) at the noted time point between species to identify expression values that were significantly different via DESeq2 (*p*-adj ≤ 0.05; |log2FoldChange|≥ 2). Notably, in both interaction analysis and cross-reference analysis, genes must be DEGs in both species between herbivory and 0 h to then be scrutinized in the second step of the comparison.

Enrichment analyses identified Gene Ontology (GO) terms enriched in various gene datasets. Over-Representation Analysis (ORA) identified GO term enrichment of DEGs. ORA was conducted via ‘enricher’ (clusterProfiler) with a post-hoc ‘gsfilter’ (DOSE) [88, 89]. Enrichment was also conducted using Kyoto Encyclopedia of Genes and Genomes (KEGG) via the KEGG Orthology-Based Annotation System-intelligent (KOBAS-i) [90, 91]. Additional information can be found in Additional Fig. 4 (Supplementary Material 1) and on GitHub at https://github.com/cdixo/Inter-species-and-Herbivory-Publication.git.

## Results

### Herbivory preference study

**Fig. 1:**
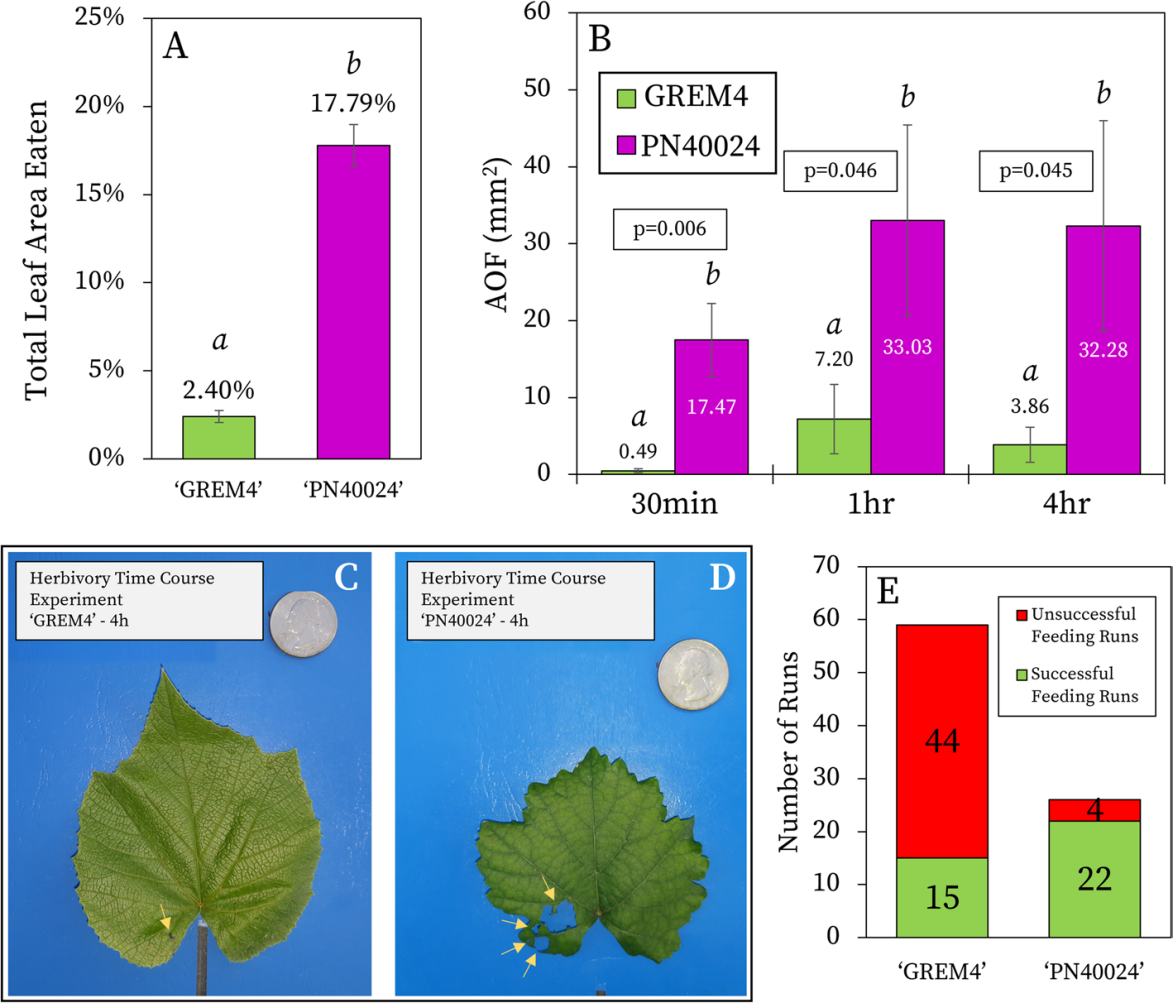
Insect herbivory studies. **A** The percentage of total leaf area eaten by Japanese beetles for ‘GREM4’ and ‘PN40024’ in the herbivory preference study, where Japanese beetles were permitted to feed upon either species *ad libitum* (*p* = 0.037; *﻿n* = 4). Error bars show the standard error. **B** Herbivory time course study average area of feeding (AOF) in mm^2^ by Japanese beetles on ‘GREM4’ and ‘PN40024’ at 30 min, 1 h, and 4 h. Significance is represented by differing letters and was calculated independently at each timepoint. Error bars show the standard error. **C** & **D** Representative images of Japanese beetle feeding damage on (**C**) ‘GREM4’ and (**D**) ‘PN40024’ mature leaves from the herbivory time course study after 4 h of feeding. Arrows indicate locations of feeding damage and a quarter was used to indicate scale. **E** Japanese beetle feeding success rate during the herbivory time course study. A run in which a Japanese beetle fed was considered ‘successful’, while a run with no feeding was ‘unsuccessful’

To determine if ‘GREM4’ was resistant to Japanese beetle herbivory, we performed a feeding preference study between ‘GREM4’ and ‘PN40024’. For all studies herein, resistance is defined as significantly decreased Japanese beetle herbivory damage on leaves from one accession compared to the other. When 15 Japanese beetles were provided the choice to feed on either ‘GREM4’ or ‘PN40024’ leaves, significantly greater herbivory damage, measured by AOF, was observed for ‘PN40024’, with 17.79% (± 1.19% S.E.) of the leaf area fed upon, compared to 2.40% for ‘GREM4’ (± 0.34% S.E.; *p* = 0.037) (Fig. 1A, Additional Fig. 6; Supplementary Material 1). These results demonstrate Japanese beetles preferred feeding on ‘PN40024’ over ‘GREM4’.

### Herbivory time course study

Next, we aimed to determine if, given no choice, Japanese beetles would still feed less on ‘GREM4’ over time, compared to ‘PN40024’. We conducted an insect herbivory time course study which restricted single Japanese beetles to either one ‘GREM4’ or one ‘PN40024’ attached leaf and allowed the beetles to feed for 30 min, 1 h, or 4 h. A significantly greater AOF was calculated for ‘PN40024’ compared to ‘GREM4’ at all time points (*﻿p*-value ≤ 0.05) (Fig. 1B-D). AOF also increased in both species from 30 min to 4 h, but little difference was observed between 1h and 4 h of feeding. These results report that, under 30 min, 1 h, and 4 h of herbivory, ‘GREM4’ experienced less AOF than ‘PN40024’, suggesting resistance to Japanese beetle herbivory.

We also recorded the number of successful (feeding) and unsuccessful (no feeding) time course runs. The majority of unsuccessful runs occurred with Japanese beetles restricted to feeding on ‘GREM4’ leaves (Fig. 1E) (‘GREM4’ = 15 successful, 44 unsuccessful runs, 25% success rate; ‘PN40024’ = 22 successful, 4 unsuccessful runs, 85% success rate). Therefore, not only was the AOF on ‘GREM4’ leaves lower, but, for the majority of the runs, the starved beetles did not feed at all. Together, these findings provide evidence that ‘GREM4’ leaves are resistant to Japanese beetle herbivory compared to ‘PN40024’.

### Leaf trichome density and herbivory studies

**Fig. 2:**
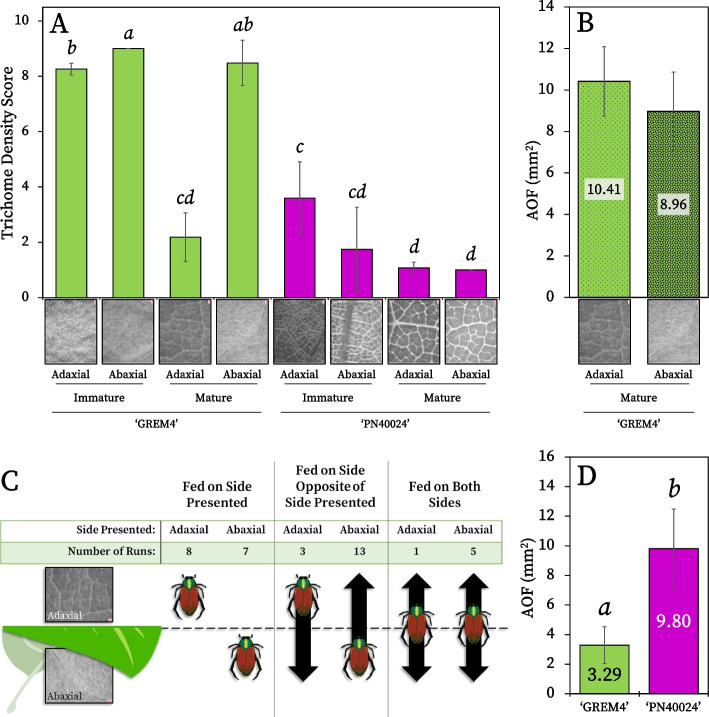
Leaf trichome density studies. In all figures, significance is denoted by differing letters above the bar graphs. The error bars denote standard errors. **A** Leaf trichome density scores. Nine images (data points) were recorded per leaf side, maturity, and species, resulting in 72 total images (*p* = < 0.001 where *n* = 9). Both ad- and abaxial sides of leaves were scored for trichome density based on the OIV ‘Mature leaf: density of prostrate hairs between main veins on lower side of blade’ scale. Representative images taken under 10 × magnification are inlayed to illustrate the differences in trichome densities. **B** Average AOF per ad- or abaxial side of ﻿‘GREM4’ leaves when trichome densities were significantly different. No significance was found (*p* = 0.307; *n* = 21 (adaxial), 10 (abaxial)). **C** Feeding preference of Japanese beetles when presented differing trichome densities in ‘GREM4’. The side of the leaf which the beetle was placed and the number of runs in which each feeding outcome occurred are reported in the table. Arrows point in the direction in which the beetles moved during the experiment. **D** Average AOF per grapevine species when trichome densities were not significantly different between the adaxial sides of the mature leaves of ‘GREM4’ and ‘PN40024’ (*﻿p* = 0.029, *n* = 10)

We next investigated the defensive mechanisms which could contribute to insect herbivory resistance in ‘GREM4’. Trichomes are a well-known insect herbivory defensive adaptation, and trichome densities visibly differed between ‘GREM4’ and ‘PN40024’ [36, 56, 57]. Therefore, we performed detailed trichome density observations on the adaxial and abaxial sides of ‘GREM4’ and ‘PN40024’ immature and mature leaves. Leaves were scored using a trichome density scale (see “Methods”), where a score of ‘1’ was devoid of trichomes while ‘9’ meant trichome density was extremely high [69]. Significantly greater trichome densities were observed in ‘GREM4’ compared to ‘PN40024’ in all comparisons, except for the adaxial side of ‘GREM4’ mature leaves (Fig. 2A). In ‘GREM4’, trichome density averages on both sides of the immature leaves and on the abaxial side of mature leaves ranged from 8.26 to 9.00, whereas the mature adaxial side was significantly less (2.19). ‘PN40024’ trichome density scores for both sides of mature and immature leaves were between 1.00 and 3.59. It should be noted that we observed ribbon and simple non-glandular trichomes on both ‘GREM4’ and ‘PN40024’ leaves, but did not observe glandular trichomes on either. These findings indicate greater trichome densities were found overall on ‘GREM4’ leaves compared to ‘PN40024’. Therefore, increased trichome density may contribute to insect herbivory resistance in ‘GREM4’.

To evaluate the impact of trichome density on ‘GREM4’ herbivory defense, we next permitted Japanese beetles to only feed on the adaxial (low trichome density) or abaxial (high trichome density) side of mature ‘GREM4’ leaves. There was no significant difference between AOF on the ad- vs. abaxial sides of the leaves (*﻿p* = 0.307) (Fig. 2B), but a feeding preference was observed during the study. Though beetles were placed on the ad- or abaxial side of the leaf, they were not completely restricted in their movement (see “Methods”). Therefore, some beetles did not feed on the presented side and instead transitioned to the non-presented side of the leaf to feed. Of beetles placed on the adaxial side of the leaf, 33% transitioned to and fed on the opposite side of the leaf (abaxial side) while 72% of beetles placed on the abaxial side transitioned to and fed on the opposite side (adaxial side) (Fig. 2C). Though the AOF was not significantly different between the two sides, these findings report that the Japanese beetles preferentially avoided the high trichome density side of the leaves, which supported the hypothesis that trichomes aid in deterring insect herbivory on ‘GREM4’.

Since trichome densities were greater on ‘GREM4’ leaves than ‘PN40024’ leaves, and considering the results from the above experiment, we additionally assessed if trichome density was the sole factor conferring heightened insect herbivory resistance in ‘GREM4’. Trichome densities on the adaxial surfaces of mature ‘GREM4’ and ‘PN40024’ leaves were not significantly different (Fig. 2A). Therefore, Japanese beetles were restricted to feed only on ‘GREM4’ and ‘PN40024’ adaxial sides of leaves. Under equal trichome density, beetles still fed about three times more on ‘PN40024’ leaves (9.80 ± 2.68mm^2^) compared to ‘GREM4’ (3.29 ± 1.25 mm^2^; *p* = 0.029) (Fig. 2D). These results suggest other factors, beyond trichomes, are also implicated in insect herbivory resistance in ‘GREM4’.

### Inter-species Transcriptomic Responses

#### Orthologous genes between ‘GREM4’ and ‘PN40024’

Orthologous genes were identified to compare transcript accumulation between ‘GREM4’ and ‘PN40024’ directly. 23,337 orthologous genes were identified between ‘GREM4’ (37,443 total annotated genes) and ‘PN40024’ (35,133) (Additional Table 2; Supplementary Material 3). An additional 12,898 ‘GREM4’ and 8,435 ‘PN40024’ paralogous genes, genes with homology with other genes in the same genome but did not have an ortholog in the other species genome (i.e. additional gene family members), were identified. This left ‘GREM4’ with 1,168 (3.12%) genome-specific genes and ‘PN40024’ with 3,321 (9.45%). The expression of orthologous genes could be compared directly between species, but genome-specific and paralogous genes could not, as they were only identified in one of the two genomes. Nonetheless, they may play important roles in conferring insect-herbivory resistance. All three categories of genes were investigated and are discussed below.

#### Basal expression differences at 0 h

**Fig. 3:**
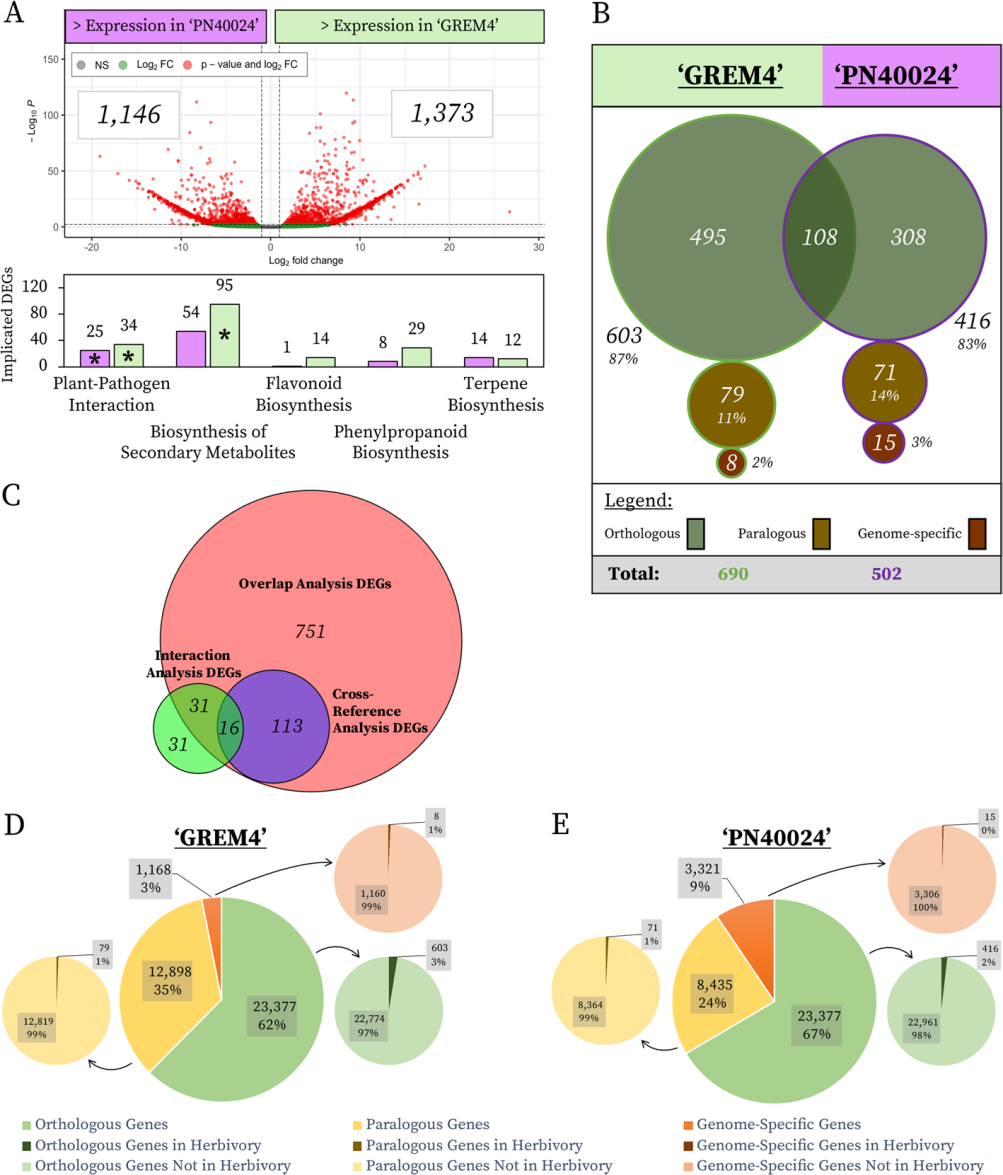
Inter-species comparisons. **A** Volcano plot of DEGs identified via DESeq2 in ‘GREM4’ compared to ‘PN40024’ under basal (0 h) conditions with a bar plot below displaying numbers of DEGs implicated in significantly enriched and other noteworthy pathways. In the volcano plot, the dashed horizontal line represents the *p*-adj threshold of ≤ 0.05 and the two dashed vertical lines denote the |log2FoldChange| threshold of ≥ 2. Dots to the right of the vertical dashed line and above the horizontal dashed line are genes which experienced statistically significantly greater expression in ‘GREM4’ compared to ‘PN40024’. Dots to the left of the vertical line and above the horizontal line are genes which experienced statistically significantly lower expression in ‘GREM4’ compared to ‘PN40024’. In the bar plot, KEGG pathway enrichments are noted along the x-axis and the number of DEGs implicated in each enrichment are noted on the y-axis. Enrichments with asterisks within the bars were significantly enriched while those without were not significantly enriched but were displayed since they are key insect herbivory defensive pathways. Green bars correspond to enrichments in genes with greater expression in ‘GREM4’ compared to ‘PN40024’, while purple bars correspond to enrichments in gene with greater expression in ‘PN40024’ compared to ‘GREM4’. **B** Diagram representing the breakdown of DEGs identified for orthologous (green), paralogous (brown), and genome-specific genes (maroon) upon insect herbivory in both ‘GREM4’ (green bordered, leftmost circles) and ‘PN40024’ (purple bordered, rightmost circles) as well as conservation between groups. Numbers of herbivory DEGs are reported, in addition to the percentages of the total number of herbivory DEGs (‘GREM4’ = 690; ‘PN40024’ = 502) in each respective species. **C** Venn diagram representing the conservation of DEGs identified using the three different inter-species orthologous gene analysis methods. **D** & **E**. Breakdown of orthologous (green palette), paralogous (yellow palette), and genome-specific genes (orange palette) implicated in herbivory responses (compared to 0 h) in ‘GREM4’ and ‘PN40024’. Small break-out pie charts display the number of DEGs identified under insect herbivory (the darker-colored small slice) out of the total genes within the group

First, expression of ‘GREM4’ and ‘PN40024’ orthologous genes at 0 h was compared to identify differences in basal expression to determine constitutively differentially expressed genes (Additional Tables 3–5; Supplementary Materials 4, 5 and 6). 1,373 of 23,377 (5.87%) orthologous genes had significantly higher expression in ‘GREM4’ compared to ‘PN40024’ at 0 h, while 1,146 (4.90%) had significantly lower expression in ‘GREM4’ compared to ‘PN40024’ (Table 1, Fig. 3A, and Additional Table 5; Supplementary Material 6). Overall, these findings indicate differences in basal transcriptomic states exist between ‘GREM4’ and ‘PN40024’.

**Table 1:** Numbers of genes identified in transcriptomic comparisons between *V. labrusca* acc. ‘GREM4’ and *V. vinifera* cv. ‘PN40024’

**Analysis**	**Species**	**Comparison**	**Expression**	**#**
**Basal Expression Differences at 0 h**	Inter-species	‘GREM4′ 0 h compared to ‘PN40024′ 0 h	Increased Expression in ‘GREM4’ Compared to ‘PN40024’	1373
			Decreased Expression in ‘GREM4’ Compared to ‘PN40024’	1146
**Insect Herbivory**	‘GREM4’	Herbivory (All Time Points Combined) compared to 0 h	Up-regulated	549
			Down-regulated	141
	‘PN40024’	Herbivory (All Time Points Combined) compared to 0 h	Up-regulated	447
			Down-regulated	55
**Overlap Analysis**	Inter-species	Unique to ‘GREM4’	Number of DEGs	495
		Unique to ‘PN40024’	Number of DEGs	308
		Conserved in both ‘GREM4’ and ‘PN40024’	Number of DEGs	108
**Interaction Analysis**	Inter-species	‘GREM4’ compared to ‘PN40024’	Increased Expression in ‘GREM4’ Compared to ‘PN40024’	45
			Decreased Expression in ‘GREM4’ Compared to ‘PN40024’	33
**Cross-Reference Analysis**	Inter-species	‘GREM4’ compared to ‘PN40024’	Number of DEGs	82
		‘PN40024’ compared to ‘GREM4’	Number of DEGs	48

Of genes with significantly higher expression in ‘GREM4’ compared to ‘PN40024’, two enriched KEGG pathways were identified (Additional Table 6; Supplementary Material 7), ‘plant-pathogen interaction’ (34 implicated DEGs) and ‘biosynthesis of secondary metabolites’ (95 implicated DEGs). Of DEGs with lower expression in ‘GREM4’ compared to ‘PN40024’ (higher expression in ‘PN40024’ at 0 h), only one pathway was enriched, ‘plant-pathogen interaction’ (25 implicated DEGs) (Additional Table 6; Supplementary Material 7). DEGs with greater expression in ‘GREM4’ compared to ‘PN40024’ at 0 h were enriched in secondary metabolite biosynthesis, which was not identified of DEGs with greater expression in ‘PN40024’ compared to ‘GREM4’ at 0 h, and likely contributed to defense against insect herbivory and other biotic stress. Though DEGs with greater expression in ‘GREM4’ and in ‘PN40024’ at 0 h were both enriched in plant-pathogen interaction, a greater number of DEGs contributed to the enrichment identified in ‘GREM4’. While a greater number of DEGs had higher expression in ‘GREM4’ at 0 h compared to ‘PN40024’, the lack of enrichment in other pathways (besides the three mentioned) suggests these genes are broadly distributed across many biological processes.

Due to their integral role in plant defense signaling [94–97], we investigated if JA and SA pathway genes were significantly differentially expressed at 0 h in ‘GREM4’ compared to ‘PN40024’. Seven JA and four SA pathway genes were significantly differentially expressed (Additional Fig. 7; Supplementary Material 1). Overall, JA and SA biosynthesis was generally skewed towards greater expression in ‘GREM4’ compared to ‘PN40024’. All seven JA DEGs and three of four SA DEGs had higher constitutive expression in ‘GREM4’ which could initiate downstream defensive pathways for heightened responses to insect and pathogen attacks.

These findings report genes implicated in defense signaling, pathogen response, and secondary metabolite biosynthesis are constitutively expressed at higher levels in ‘GREM4’ compared to ‘PN40024’ and may contribute to the increased insect herbivory resistance.

#### Insect herbivory

The total number of DEGs at 30 min, 1 h, and 4 h of Japanese beetle herbivory (compared to 0 h) were determined for ‘GREM4’ and ‘PN40024’ and combined between time points (duplicates removed). A total of 690 (549 up-regulated and 141 down-regulated) DEGs were identified in ‘GREM4’ under herbivory, while a total of 502 (447 up-regulated and 55 down-regulated) DEGs were identified in ‘PN40024’ (Table 1), thus, more genes were differentially expressed in ‘GREM4’ under herbivory compared to ‘PN40024’. These identified DEGs could have been orthologous, paralogous, or genome-specific, which we investigated below.

We first identified genes with significantly different transcript accumulation between ‘GREM4’ and ‘PN40024’ upon Japanese beetle herbivory (Additional Tables 3–5; Supplementary Materials 4, 5 and 6). We conducted these analyses via three methods — ‘Overlap Analysis’, ‘Interaction Analysis’, and ‘Cross-Reference Analysis’ (see ﻿“Methods﻿” and Additional Fig. 5; Supplementary Material 6) — to identify DEGs, and identify candidate genes, likely contributing to increased insect herbivory resistance in ‘GREM4’.

##### Overlap analysis

Overlap analysis identified orthologous genes which were significantly up or down-regulated under herbivory compared to 0 h at any time point in both species. Out of the total 1,192 DEGs identified under insect herbivory in ‘GREM4’ and ‘PN40024’, 911 had orthologs in both genomes. Of these 911 orthologs, only 108 DEGs were significantly differentially expressed in both ‘GREM4’ and ‘PN40024’ (Fig. 3B and Additional Table 7; Supplementary Material 8) and overlapping DEGs were enriched for genes involved in ‘sequence-specific DNA binding’ (Additional Table 8; Supplementary Material 9). 495 of the orthologous genes were only differentially expressed in ‘GREM4’ (Fig. 3B and Additional Table 7; Supplementary Material 8) and ORA enrichment analysis revealed ‘hydrolase activity, acting on ester bonds’ as the only functional enrichment for these DEGs (Additional Table 8; Supplementary Material 9). Nonetheless, genes implicated in other pathways were identified in this list as well, including lipid formation, terpene biosynthesis, and peroxidase activity. 308 orthologs were only differentially expressed in ‘PN40024’ (Fig. 3B and Additional Table 7; Supplementary Material 8) and nine functional enrichments were identified including xyloglucan-related terms, ‘cell wall biogenesis’, and ‘calcium ion binding’ (Additional Table 8; Supplementary Material 9). These results report that, although the majority of differentially expressed genes under herbivory were orthologous, only about 12% were significantly differentially expressed in both species, suggesting specialized expression patterns for the majority of these orthologous DEGs in each species under insect herbivory.

##### Interaction analysis

The interaction analysis identified orthologous genes with a significant Δlog2FoldChange between ‘GREM4’ and ‘PN40024’. Out of 23,377 orthologous genes, only 78 had a significant Δlog2FoldChange between ‘GREM4’ and ‘PN40024’ and 58% of these 78 had a greater Δlog2FoldChange in ‘GREM4’ compared to ‘PN40024’ (Table 1, Additional Tables 5 and 7; Supplementary Materials 6 and 8). The top 10 genes that were identified via the interaction analysis had a |Δlog2FoldChange|≥ 20 and a *p*-adj ≤ 0.01 and were implicated in terpene biosynthesis, disease and pathogen resistance, and wax biosynthesis (Table 2 and Additional Table 6; Supplementary Material 7). Eight of these ten genes had greater Δlog2FoldChange in ‘GREM4’ and are candidate genes for insect herbivory resistance and future study.

**Table 2:** *V. labrusca* acc. ‘GREM4’ candidate insect herbivory resistance genes from the interaction analysis

**#**	⥮	**Biological Implication**	**Abbreviated Gene Name**	***V. labrusca* acc. ‘GREM4’ Gene**	**Full Gene Name**
1	**↑**	**Terpene Biosynthesis**	*BAS isoform X2/CAMS1*	*Vitla_GREM4_10g108.60*	*Beta-amyrin synthase isoform X2 / Camelliol C synthase 1*
2	**↑**	**Putative Pathogen Resistance**	*RPS2*	*Vitla_GREM4_12g237.26*	*Putative Resistant to P. syringae 2*
3	**↓**	**Disease Resistance; SAR and ETH Induction**	*GLIP2*	*Vitla_GREM4_10g58.5*	*GDSL esterase/lipase 2*
4	**↑**	**Phosphate Transport**	*PHO1-like 3*	*Vitla_GREM4_1g132.33*	*Phosphate 1-like 3*
5	**↓**	**Wax Biosynthesis**	*CER1/22*	*Vitla_GREM4_15g100.37*	*Eceriferum 1/22*
6	**↑**	**Disease Resistance**	*RPP13-like*	*Vitla_GREM4_13g144.42*	*Putative disease resistance RPP13-like*
7	**↑**	**Disease Resistance**	*PR1-like 1*	*Vitla_GREM4_3g126.4*	*Pathogenesis-related protein 1-like 1*
8	**↑**	**Disease Resistance**	*N-like 1*	*Vitla_GREM4_00g37057*	*TMV resistance protein N-like protein 1*
9	**↑**	**Terpene Biosynthesis**	*TPS1-like*	*Vitla_GREM4_19g14.9*	*Terpene synthase 1-like*
10	**↑**	**-**	*-*	*Vitla_GREM4_00g74.30*	*-*

‘- ‘ indicates that no functional annotation was identified for the gene via our annotation pipeline

##### Cross-reference analysis

The cross-reference analysis identified orthologous genes which were significantly differentially expressed during herbivory compared to 0 h and had significantly different expression between ‘GREM4’ and ‘PN40024’ under herbivory (read count value) at the coincidental time point. When combining all up and down-regulated DEGs across all time points, 82 such genes were identified in ‘GREM4’ compared to ‘PN40024’ (Table 1 and Additional Table 7; Supplementary Material 8). Comparatively, in ‘PN40024’, only 48 genes were identified under the same parameters (Table 1 and Additional Table 7; Supplementary Material 8). Of the 82 ‘GREM4’ genes, the top 12 had a |log2FoldChange|≥ 20 and a *p*-adj ≤ 0.01 (Table 3). These 12 DEGs were implicated in phytohormonal response, disease/fungal resistance, terpene biosynthesis, and flavonoid biosynthesis and are candidate genes for insect herbivory resistance and future study.

**Table 3:** *V. labrusca* acc. ‘GREM4’ candidate insect herbivory resistance genes from the cross-reference analysis

**#**	⥮	**Biological Implication**	**Abbreviated Gene Name**	***V. labrusca* acc. ‘GREM4’ Gene**	**Full Gene Name**
1	**↑**	**-**	*-*	*Vitla_GREM4_14g4.6*	-
2	**↑**	**Response to SA; Cell Wall Formation**	*GRP5-like 1*	*Vitla_GREM4_7g96.2*	*Glycine rich protein 5-like 1*
3	**↑**	**Response to SA; Cell Wall Formation**	*GRP5-like 2*	*Vitla_GREM4_7g95.10*	*Putative Glycine rich protein 5-like 2*
4	**↓**	**Disease Resistance; SAR and ETH Induction**	*GLIP2*	*Vitla_GREM4_10g58.5*	*GDSL esterase/lipase 2*
5	**↑**	**Phytohormone Regulation; Antioxidant and Defense Metabolite Biosynthesis**	*CYP-like*	*Vitla_GREM4_15g170.54*	*Cytochrome P450-like*
6	**↑**	**JA Biosynthesis**	*AOS3*	*Vitla_GREM4_3g53.38*	*Allene oxide synthase 3*
7	**↑**	**Fungal Defense; Glucosinolate Processing**	*BGLU16*	*Vitla_GREM4_13g317.61*	*Beta glucosidase 16*
8	**↑**	**-**	*-*	*Vitla_GREM4_5g213.11*	*-*
9	**↑**	**Disease Resistance; SAR and ETH Induction**	*GLIP1*	*Vitla_GREM4_19g86.34*	*GDSL esterase/lipase 1*
10	**↑**	**Pectin Cell Wall Remodeling; Pathogen Resistance**	*PMEI25*	*Vitla_GREM4_13g203.16*	*Pectin methylesterase inhibitor 25*
11	**↑**	**Terpene Biosynthesis**	*CYP716A1*	*Vitla_GREM4_18g311.31*	*Cytochrome P450 monooxygenase, family 716, subfamily A, polypeptide 1 / Beta-amyrin 28-monooxygenase-like*
12	**↑**	**Flavonoid Biosynthesis**	*F3H*	*Vitla_GREM4_4g210.29*	*Flavanone 3-hydroxylase*

‘- ‘ indicates that no functional annotation was identified for the gene via our annotation pipeline

Overall, all three comparative methods reported genes implicated in processes and pathways related to insect herbivory defense and some genes (Tables 2 and 3) have been identified as potential candidates for future insect herbivory resistance functional validation. These three methods cooperatively identified genes of interest by either capturing genes overlooked, or refining a pool identified, by another method (Fig. 3C). Sixteen DEGs were captured by all three methods and were thus very strong candidates to confer insect herbivory resistance (Fig. 3C and Table 4). These 16 genes were implicated in disease resistance, insect herbivory resistance and response, biotic stress response, JA and SA, pollen-related functions, and photosynthesis under stress.

**Table 4:** *V. labrusca* acc. ‘GREM4’ insect herbivory genes identified by all three transcriptomic comparison methods

#	Biological Implication	Abbreviated Gene Name	*V. labrusca* acc. ‘GREM4’ Gene	Full Gene Name
1	**Disease Resistance; SAR and ETH Induction**	*GLIP2*	*Vitla_GREM4_10g58.5*	*GDSL esterase/lipase 2*
2	-	*-*	*Vitla_GREM4_14g4.6*	*-*
3	**Response to SA; Cell Wall Formation**	*GRP5-like 1*	*Vitla_GREM4_7g96.2*	*Glycine rich protein 5-like 1*
4	**Pollen Grain Compatibility**	*RKFL1*	*Vitla_GREM4_10g56.41*	*Receptor-like kinase in flowers 1*
5	**Biotic Stress Response**	*HSP*	*Vitla_GREM4_13g82.26*	*Class I heat shock protein*
6	**Cuticular Wax Formation**	*MAH1*	*Vitla_GREM4_14g270.32*	*Mid-chain alkane hydroxylase 1*
7	**Photosynthesis Under Senescence and High-Light**	*FTSH6*	*Vitla_GREM4_14g293.28*	*FTSH protease 6*
8	**Pathogen Resistance; Abiotic Stress Tolerance; Plant Development**	*BAG6*	*Vitla_GREM4_15g196.45*	*BCL-2-associated athanogene 6*
9	**Possible Implication in Flavonoid Biosynthesis/Insect Resistance**	*UGT88A1*	*Vitla_GREM4_16g200.49*	*UDP-glucosyl transferase 88A1*
10	**SA/MeSA Regulation**	*SAMT2*	*Vitla_GREM4_00g36975*	*Salicylate carboxymethyl transferase 1*
11	-	*-*	*Vitla_GREM4_4g0.9*	*-*
12	**ER-related; Intra-cellular Transport; Ion Transport**	*ER body-like protein*	*Vitla_GREM4_00g188.10*	*ER body-like protein*
13	**Biotic Stress Response**	*HSP-2*	*Vitla_GREM4_8g87.15*	*Class I heat shock protein - 2*
14	**Protein Binding**	*XIAO*	*Vitla_GREM4_9g148.35*	*Putative inactive leucine-rich repeat receptor kinase XIAO*
15	**Insect Herbivory Resistance; JA and JA-Ile Biosynthesis; Pollen Chemi-attractance**	*MIK2*	*Vitla_GREM4_13g250.49*	*MDIS1-interacting receptor like kinase 2*
16	**Pathogen Resistance; Insect Herbivory Response**	*RLP27-like*	*Vitla_GREM4_8g145.10*	*Receptor like protein 27-like*

‘- ‘ indicates that no functional annotation was identified for the gene via our annotation pipeline

### Functions of genome-specific and paralogous genes

The inter-species analyses conducted above only compared gene expression differences under herbivory for genes with an ortholog in both species. But genome-specific and paralogous genes, for which a direct ortholog could not be identified, are also of interest since they can be major contributors to genetic novelty.

Genes which were only identified in ‘GREM4’ or ‘PN40024’ were identified as ‘genome-specific genes’. In ‘GREM4’, 1,168 genome-specific genes were identified (Fig. 3D, Additional Tables 2 and 7; Supplementary Material 3 and 8), and while no functional enrichments via ORA were identified (Additional Table 8; Supplementary Material 9), one KEGG pathway was enriched of ‘plant-pathogen interactions’ (34 genes) (Additional Table 6; Supplementary Material 7). This result suggests some ‘GREM4’ genome-specific genes contribute to interactions with pathogens, but the rest are distributed across a myriad of metabolic pathways, with, for example, 9% being found to be involved specifically in secondary metabolite biosynthesis. Of the 690 total DEGs identified in the ‘GREM4’ herbivory samples compared to 0 h, only eight (2%) were genome-specific (Fig. 3B, Table 5) representing < 1% of all genome-specific genes in ‘GREM4’ (Fig. 3D). In ‘PN40024’, 3,321 genome-specific genes were identified (Fig. 3E, Additional Tables 2 and 7; Supplementary Material 3 and 8), but while no KEGG pathways were significantly enriched (Additional Table 6; Supplementary Material 7), one functional enrichment was identified, ‘cytochrome complex assembly’ (Additional Table 8; Supplementary Material 9). This result suggests ‘PN40024’ genome-specific genes, like ‘GREM4’, contribute to a broad range of functions and pathways. Of the 502 total ‘PN40024’ herbivory DEGs, only 15 (3%) were genome-specific (Fig. 3B), representing < 1% of all genome-specific genes (Fig. 3E).

**Table 5:** *V. labrusca* acc. ‘GREM4’ paralogous and genome-specific candidate insect herbivory resistance genes

Biological Implication	Abbreviated Gene Name	*V. labrusca* acc. ‘GREM4’ Gene	Full Gene Name	Gene Group
**Reactive Oxidative Species**	*LOX1*	*Vitla_GREM4_6g20.0*	*Lipoxygenase 1*	*Genome-specific*
**Stigmasterol Biosynthesis**	*CYP710A11*	*Vitla_GREM4_10g73.23*	*Cytochrome P450 monooxygenase, family 710, subfamily A, polypeptide 11*	*Genome-specific*
**Reactive Oxidative Species**	*Predicted protein HHK36*	*Vitla_GREM4_14g248.28*	*Predicted protein HHK36 (Peroxidase)*	*Genome-specific*
**MeJA Conversion to JA**	*MJE1*	*Vitla_GREM4_00g37214*	*Methyl jasmonate esterase 1*	*Genome-specific*
-	*-*	*Vitla_GREM4_11g38.27*	*-*	*Genome-specific*
-	*-*	*Vitla_GREM4_18g79.1*	*-*	*Genome-specific*
-	*-*	*Vitla_GREM4_6g24.28*	*-*	*Genome-specific*
-	*-*	*Vitla_GREM4_13g88.14*	*-*	*Genome-specific*
**Wax Biosynthesis; Development**	*WSD1*	*Vitla_GREM4_12g40.2*	*O-methyltransferase (Wax synthase/acyl-CoA:diacylglycerol acyltransferase)*	*Paralogous*
**Disease Resistance; SAR and ETH Induction**	*GDSL-like*	*Vitla_GREM4_18g317.26*	*GDSL-like lipase/Acylhydrolase*	*Paralogous*

‘- ‘ indicates that no functional annotation was identified for the gene via our annotation pipeline

Next, we investigated paralogous genes (e.g. extra gene copies unique to a species). 12,898 paralogous genes were detected in ‘GREM4’ (Fig. 3D, Additional Tables 2 and 7; Supplementary Material 3 and 8) and were enriched in 30 functional enrichments including ‘signal transduction’, ‘lignin catabolic process’, terpene-related terms, acyltransferase-related terms, and ‘transcription coactivator activity’ (Additional Table 8; Supplementary Material 9). Of the 690 herbivory DEGs in ‘GREM4’, 79 (11%) were paralogous genes (Fig. 3B), representing < 1% of all paralogous genes in ‘GREM4’ (Fig. 3D). Four functional enrichments were identified in these 79 genes: ‘signal transduction’, ‘biosynthetic process’, and two acyltransferase-related terms (Additional Table 8; Supplementary Material 9). When identifying the topmost significantly differentially expressed genes via the parameters |log2FoldChange|≥ 20 and a *p*-adj ≤ 0.01, two ‘GREM4’ herbivory DEGs were paralogs (Table 5). In ‘PN40024’, 8,435 paralogous genes were identified (Fig. 3E, Additional Tables 2 and 7; Supplementary Material 3 and 8) and were enriched in 35 ORA functional terms including ‘DNA integration’, cellulose-related terms, and ‘response to auxin’ (Additional Table 8; Supplementary Material 9). 71 (14%) of the total 502 ‘PN40024’ herbivory DEGs were paralogous genes (Fig. 3B), representing < 1% of all paralogous genes (Fig. 3E). ‘Apoplast’ was the only functional enrichment in these 71 genes (Additional Table 8; Supplementary Material 9). These results report ‘GREM4’ had a greater number of paralogous genes, indicating more gene family expansions, and/or fewer gene family contractions, compared to ‘PN40024’, and a portion of those genes were differentially expressed under insect herbivory, suggesting a role in defense response.

Gene family expansions can give rise to genes with novel or specialized functions, expression patterns, or activity. To explore how such genes could impact insect herbivory defense, we investigated two gene families which differ in gene family size between ‘GREM4’ and ‘PN40024’ and displayed significantly different expression upon Japanese beetle herbivory. The *TPS1*-orthogroup gene family was identified via OrthoFinder and is implicated in terpene biosynthesis. The *TPS1*-orthogroup gene family differs in gene family members between ‘PN40024’ (four genes) and ‘GREM4’ (eight genes) and two genes unique to ‘GREM4’, Terpene synthase 1–2 (*TPS1-2*) (*Vitla_GREM4_19g60.31*) and Terpene synthase 1–3 (*TPS1-3*) (*Vitla_GREM4_19g59.46*), experienced increased expression upon beetle herbivory (Additional Fig. 8A; Supplementary Material 1). As for constitutive expression, *TPS1-3* also displayed the highest expression (read count value) of any family member in ‘GREM4’ at 0 h (Additional Table 3; Supplementary Material 4). The second gene family explored was Phenylalanine lipase (*PAL*), genes encoding enzymes that catalyze the reaction converting phenylalanine to cinnamic acid in the phenylpropanoid pathway, which is critical to both flavonoid and lignin biosynthesis [98]. Four gene family members were identified in the *PAL* gene family in ‘PN40024’ while 12 were identified in ‘GREM4’, four of which were differentially expressed upon insect herbivory — *PAL1-4* (*Vitla_GREM4_16g7.31*), *PAL1-5* (*Vitla_GREM4_16g8.34*), *PAL1-6* (*Vitla_GREM4_16g8.37*), and *PAL1-8* (*Vitla_GREM4_16g7.35*) (Additional Fig. 8B; Supplementary Material 1). When reviewing constitutive expression, ‘GREM4’ novel gene *PAL1-11* (*Vitla_GREM4_8g123.37*) was expressed hundreds of times more than most other *PAL* genes at 0 h (Additional Table 3; Supplementary Material 4). For example, *PAL1-11* had a read count value of 7,415, whereas *PAL1-4* had a read count value of 1,045 and *PAL1-6* had a value of 9. These results suggest that *PAL* and *TPS* paralogous genes unique to ‘GREM4’ are involved in a response to insect herbivory, and in some cases, are constitutively expressed at high levels. It is likely these genes are important in conferring heightened insect herbivory defense via terpene, flavonoid, lignin, or other phenolic compound production.

Though some paralogous (11% and 14%) and genome-specific (2% and 3%) genes were differentially expressed under herbivory in both ‘GREM4’ and ‘PN40024’, it is striking that 87% and 83% of the DEGs during herbivory in ‘GREM4’ and ‘PN40024’ were orthologous genes (Fig. 3B). Additionally, only 108 of the 603 ‘GREM4’ and 416 ‘PN40024’ total orthologous herbivory DEGs were differentially expressed during herbivory in both species, suggesting differential expression of orthologous genes is key in imparting insect herbivory resistance (Fig. 3B).

Taken together, these findings suggest that the heightened insect herbivory resistance of ‘GREM4’ compared to ‘PN40024’ is greatly due to unique expression patterns of orthologous genes in ‘GREM4’ and, to a lesser degree, expression of paralogous and genome-specific genes involved in plant-pathogen interactions and secondary metabolism. Additional functional studies are necessary to fully elucidate the impact of paralogous and genome-specific insect herbivory response candidate genes outlined in Table 5.

## Discussion

Plants are sessile organisms, so the evolution of defensive measures to counteract threats, including insect herbivory, is essential for survival and reproduction. Defenses against herbivory are diverse and include trichomes, lignified tissue, thick waxy cuticles, chemical defenses such as insecticidal or repellent secondary metabolites, and volatile organic compound signaling [47, 53, 59, 99–101]. *V. labrusca* is commonly used in grapevine breeding programs to instill resistance to biotic and abiotic stresses, but the underlying contributors to this resistance are not well understood. In this study, we evaluated *V. labrusca* acc. ‘GREM4’ and *V. vinifera* cv. ‘PN40024’ for herbivory resistance against Japanese beetle, determined the role of trichomes in herbivory defense, and identified genes involved in responses to insect herbivory*.*

### ‘GREM4’ is resistant to Japanese beetle herbivory

‘GREM4’ exhibited increased Japanese beetle herbivory resistance compared to ‘PN40024’ in both our choice and no-choice experiments, across multiple feeding time points. Our results support and expand upon previous reports that *V. labrusca-*hybrid grapevines exhibited decreased Japanese beetle herbivory compared to *V. vinifera* [23]. Past studies have also reported heightened insect herbivory resistance in other North American wild grapevines; A screen of North American grapevine species and hybrid *Vitis* cultivars for mealybug resistance found *V. vinifera* lines were highly infested with mealybugs, while North American hybrids experienced little infestation [38]. Insect herbivory resistance has been widely identified in wild relatives of other crops, such as wild soybean (*Glycine soja*), exotic cotton landraces (*Gossypium hirsutum*), and maize landraces (*Zea mays*) [102–104]. Wild plant species/accessions often possess heightened resistance to biotic and abiotic stress, and consequently, have long been employed in breeding programs as sources of novel genetic material to imbue advantageous traits to elite lines [34, 36]. In general, *V. labrusca* is highly fit in its local environment against pathogens and adverse weather conditions [27–30, 32, 33], so it was not surprising our herbivory experiments supported our hypothesis that *V. labrusca* accession ‘GREM4’ was more resistant to Japanese beetles herbivory compared to ‘PN40024’.

### Trichome density contributes to insect herbivory resistance

Since trichomes are well-known plant adaptations that aid in defense against insect herbivory [36, 56, 57], we tested whether increased trichome density was responsible for conferring heightened insect herbivory resistance in ‘GREM4’ compared to ‘PN40024’. Our results determined that leaf trichome densities were significantly greater on ‘GREM4’ leaves compared to ‘PN40024’, which is consistent with previous ampelographic studies of trichomes in *Vitis* [18, 31, 92, 93]. When beetles were placed on high trichome density sides of ‘GREM4’ leaves, they moved to the low trichome density side of the leaf to feed 72% of the time, suggesting trichomes deter Japanese beetle herbivory.

The impact of trichomes on insect defense in crop plants is well established. For example, high trichome densities have resulted in decreased insect damage in wheat, *Datura stramonium*, and soybean [56, 58, 59]. Trichomes appear to contribute to insect herbivory defense in grapevines, however, insect size and mouthpart type seem to determine their effectiveness. In interspecific grapevine ‘GE1025’ for example, a weak negative correlation was identified between phylloxera severity traits, phylloxera being a small piercing-sucking mouthpart insect, and trichome density of leaves [105]. Anecdotally, *V. labrusca* hybrid ‘Edelweiss’ exhibited decreased phylloxera damage due to its high trichome density, as well [105]. Large, chewing mouthpart insects, such as Japanese beetles, are most deterred by high trichome densities as the trichomes can physically obstruct the insects from accessing the plant tissues beneath to feed, as is seen in other species [59]. This phenomenon was observed by Johnson et al., where *V. vinifera* acc. ‘Mars’, with high trichome density, had the least amount of Japanese beetle feeding damage in a *V. vinifera* panel [106], supporting previous findings [21]. Our results also support Japanese beetle herbivory is deterred by the high trichome density of ‘GREM4’.

Besides serving as a physical barrier to herbivory, glandular trichomes can secrete secondary metabolites or mucilage to deter or trap insect herbivores [93]. In our study, we did not observe glandular trichomes on the grapevine leaves. This supports previous reports of a general lack of glandular trichomes in *Vitis*. However, some studies have identified glandular trichomes, at low and variable densities, on *V. labrusca* branchlets and petioles*,* but not on *V. vinifera* [92, 93]. Glandular trichomes may be infrequent in *V. labrusca* and could be accession specific, with ﻿‘GREM4﻿’ representing an accession that lacks them. Since we did not observe glandular trichomes on ‘GREM4’ leaves, we suspect the high trichome density acts as a physical barrier to deter Japanese beetle feeding.

Importantly, in our study when Japanese beetles were strictly allowed to feed on mature adaxial sides of ‘GREM4’ and ‘PN40024’ leaves with similarly low trichome densities, there was still significantly less (~ 3 times less) AOF in ‘GREM4’ than ‘PN40024’. This finding indicates trichomes are not the only factor contributing to herbivory resistance in ‘GREM4’.

### Defense response genes are constitutively expressed at higher levels in ‘GREM4’ compared to ‘PN40024’

Constitutive defense in plants is a phenomenon where defensive structures, compounds, etc. are always produced or present, even when the stress is not experienced, to provide an immediate level of protection when encountered [107]. In our study, a comparison between basal transcript accumulation levels of ‘GREM4’ (0 h) compared to ‘PN40024’ (0 h) revealed 2,519 DEGs between the two species.

DEGs with greater expression in ‘GREM4’ compared to ‘PN40024’ were enriched in pathways involved in ‘plant-pathogen interaction’ and ‘biosynthesis of secondary metabolites’. It is not uncommon for genes implicated in pathogen interaction and resistance to be differentially expressed under insect herbivory, as they often serve multiple roles in biotic stress response, including insect herbivory, in a variety of plants including grapevine [57, 108–112]. Considering *V. labrusca* is resistant to many pathogens we propose it is likely these genes play a role in conferring heightened constitutive defense against pathogens, as well as insects, in ‘GREM4’ [29, 30, 32, 33]. While ‘plant-pathogen interactions’ was also an enrichment term for genes with higher constitutive expression in ‘PN40024’, a greater number of such genes, and different genes, were more highly expressed in ‘GREM4’. Specialized expression of different plant-pathogen interaction orthologous genes in response to herbivory has evolved in each species.

Additionally, DEGs with constitutively increased expression in ‘GREM4’ compared to ‘PN40024’ were enriched in genes involved in the ‘biosynthesis of secondary metabolites’ pathway and the implicated 149 genes were mainly associated with terpene, carotenoid, phenylalanine-tyrosine-tryptophan, flavone-flavanol, stilbenoid, and flavonoid biosynthesis. Considering the role of terpenes, flavonoids, and other secondary metabolites in insect herbivory defense [47–49, 51, 54, 61, 113, 114], it seems likely that increased basal expression of these genes translates to increases in such metabolites, conferring heightened constitutive defense against insect herbivory, though metabolomic tests are required to verify this hypothesis.

Heightened constitutive expression resulting in insect defense has been observed in other species. Sitka spruce (*Picea sitchensis*) genotypes with resistance to spruce weevil (*Pissodes strobi*) constitutively expressed over 2,000 genes at greater levels, and had twice as many constitutively expressed genes associated with defense-related GO terms, than susceptible genotypes prior to insect herbivory [115]. In alfalfa (*Medicago sativa*) resistant to thrips, when compared to a susceptible line under non-insect herbivory conditions, the resistant line had higher levels of flavonoid compounds [116]. In wheat (*Triticum aestivum*), a variety resistant to maize weevil (*Sitophilus zeamais*) constitutively produced multiple compounds, including flavonoids and benzoxazinoids, at levels greater than the susceptible variety [52].

Overall, the high constitutive expression of defense genes in ‘GREM4’ relative to ‘PN40024’ likely provides greater immediate defense against Japanese beetle herbivory.

### Unique genes and gene expression are implicated in ‘GREM4’ insect herbivory defense

In our study, the majority of genes which were differentially expressed under herbivory were orthologous between ‘GREM4’ and ‘PN40024’. This result suggests differences in gene regulation between species is a crucial factor in conferring heightened insect herbivory resistance in ‘GREM4’ compared to ‘PN40024’.

Previous genomic studies have reported extensive structural differences between ‘GREM4’ and ‘PN40024’, likely impacting its fitness [60]. Structural variations, including duplications, insertions, and deletions, as well as small indels and SNPs, impact gene content, gene zygosity, and gene regulation between ‘GREM4’ and ‘PN40024’ [60]. This genetic variation between ‘GREM4’ and ‘PN40024’ in genic and regulatory regions likely contributed to the observed differential expression of orthologous genes under insect herbivory in our study, through the modification or degeneration of cis-regulatory elements, or the transcripts themselves, leading to differential defense responses.

Genome-specific and paralogous genes are unique to a species, often exhibiting novel or specialized functions or regulation that give rise to distinctive phenotypes. Genome-specific and paralogous genes were found to comprise a relatively small percentage of total DEGs upon herbivory in our study (13% in ‘GREM4’ and 17% in ‘PN40024’), but, nonetheless, play a role in insect herbivory defense.

Segmental duplications result in paralogous genes and were reported as key drivers of genome evolution and diversification in ‘GREM4’ by contributing to the rapid amplification of gene families involved in environmental response, including defense response [60]. Our study found paralogous genes in ‘GREM4’ contributed to insect herbivory defense responses. For example, *PAL1,* an enzyme critical to the phenylpropanoid pathway, had 12 gene copies in ‘GREM4’, but only four in ‘PN40024’, and the *TPS1-*orthogroup gene family, implicated in terpene biosynthesis, had eight gene copies in ‘GREM4’, but only four in ‘PN40024’. These novel paralogs had heightened expression upon insect herbivory in ‘GREM4’, likely resulting in increased flavonoid and/or lignin and terpene production, in turn increasing defense. Metabolomic analysis is necessary to confirm this connection. These results further support the premise that duplicated genes impact responses to environmental stress and contribute to increased plant fitness.

Examples of gene family expansions playing a role in insect herbivory resistance have been observed in other species. Threonine deaminase (*TD1*), a gene which encodes an enzyme critical in the formation of isoleucine, is an example of a gene duplication event resulting in a paralog with novel function. *TD2* (the paralog of *TD1* in tomato) had lower isoleucine biosynthetic capacity compared to *TD1*, but uniquely impaired insect digestion, while *TD1* significantly increased in expression upon MeJA application and wounding [117, 118]. Another example is the expansion of the Lipoxygenase (*LOX*) gene family, which is important in various biological processes including ROS, JA, and defense [119]. In wheat, 44 *LOX* gene family members were identified compared to only 6–13 in other plants [120]. After 48-72 h of English grain aphid herbivory in a resistant genotype, *LOX5, LOX7, LOX10, LOX24, LOX29,* and *LOX33* were up-regulated but had lower expression in a susceptible genotype [120]. These studies provide support that gene family paralogs can exhibit differential responses to insect herbivory compared to other family members and contribute to resistance.

Overall, our study reports paralogous and genome-specific genes in ‘GREM4’ likely play a role in conferring insect herbivory resistance. However, altered expression of orthologous genes, which constituted the majority of DEGs under herbivory, appear to be the major contributors.

### Phytohormone signaling, secondary metabolite, and pathogen response pathways implicated in ‘GREM4’ insect herbivory response

The DEGs involved in defense responses to insect herbivory in ‘GREM4’ were especially enriched in functions related to secondary metabolite biosynthesis, phytohormone signal transduction, and pathogen defense and play a role in conferring the heightened insect herbivory resistance observed in ‘GREM4’.

Phytohormones are critical signaling molecules essential to plant development, stress response, and insect herbivory defense [94–97]. In our study, we identified multiple DEGs under herbivory involved in ethylene (ETH), SA, and JA biosynthesis and regulation, many of which were highly expressed in ‘GREM4’ compared to ‘PN40024’. Alterations in phytohormone accumulations signal downstream defense responses, such as secondary metabolite biosynthesis.

Secondary metabolites are key defensive compounds produced by plants in response to insect herbivory. The pathway ‘biosynthesis of secondary metabolites’ was enriched in both ‘GREM4’ and ‘PN40024’ herbivory responses, but, in ‘GREM4’, a greater number of DEGs (87 compared to 48) were associated with this pathway. ‘Biosynthesis of secondary metabolites’ was also enriched in DEGs with greater expression in ‘GREM4’ compared to ‘PN40024’ under basal conditions. Genes associated with secondary metabolite biosynthesis were also identified via overlap analysis, interaction analysis, and cross-reference analysis and were identified as candidate genes (see Tables 2– 5).

Terpenes are a class of secondary metabolites which contribute to insect herbivory resistance in plants, as well as play roles in flavor, signaling, and development [46, 114, 121–124]. Insect herbivory of leaves revealed enrichment of DEGs implicated in terpene-related functions and pathways in ‘GREM4’, but not in ‘PN40024’. Interaction analysis and cross-reference analysis revealed terpene biosynthesis genes as some of the topmost significantly differentially expressed genes upon insect herbivory, likely contributing increased insect herbivory resistance in ‘GREM4’.

Terpenes have been reported to play roles in insect herbivory resistance in grapevine and other crops. In *V. labrusca* x *V. riparia* hybrid ‘Beta’, volatile terpene production increased in the days following Japanese beetle herbivory of leaves [113]. Genes implicated in terpene biosynthesis also undergo expression alterations in response to insect herbivory. *TPS* genes, which are implicated in terpene biosynthesis, for example, were up-regulated in rice (*Oryza sativa*) upon Asiatic rice borer (*Chilo suppressalis*) herbivory and in tea (*Camellia sinensis*) upon tea geometrid (*Ectropis obliqua*) feeding [48, 121]. Additionally, D-limonene synthase (a terpene biosynthesis gene) maize mutants exhibited increased corn borer damage, reinforcing the importance of terpene genes in insect herbivory defense [114]. Downstream analyses are necessary to determine if considerations such as the quantity or unique activities of terpenes produced in ‘GREM4’ impart the heightened insect herbivory resistance.

Flavonoids are widely recognized as insect herbivory defensive compounds in plants and are broadly insecticidal [49, 51, 53, 54]. In our study, genes involved in the flavonoid biosynthesis pathway were exclusively enriched in ‘GREM4’ leaf herbivory DEGs compared to ‘PN40024’. Increased expression of genes implicated in flavonoid biosynthesis and accumulation have been observed upon insect herbivory, as seen with oriental longheaded grasshopper herbivory of *V. vinifera* x *V. labrusca* hybrid ‘Kyoho’ [61]. Flavonoids have also been documented as insect herbivory defense compounds, as flavonoids extracted from sorghum were insecticidal to fall armyworm (*Spodoptera frugiperda*) and increased mortality was observed when feeding upon maize overproducing flavonoids compared to wild-type lines [51]. In insect herbivory resistant rice, flavonoid accumulations significantly increased upon brown planthopper (*Nilaparvata lugens*) feeding, but significantly decreased in a susceptible cultivar [49]. These results suggest flavonoids are likely key contributors in conferring heightened insect herbivory resistance in ‘GREM4’. Future metabolomic analyses are necessary to validate this finding.

Genes implicated in disease resistance, pathogen response, plant-pathogen interactions, and other related processes and pathways, were widely implicated in ‘GREM4’ insect herbivory responses but were not as prominently observed in ‘PN40024’ in our study. Pathogen defense-related genes were enriched in ‘GREM4’ DEGs compared to ‘PN40024’ under basal conditions, as well as in the overlap analysis, interaction analysis, and cross-reference analysis. Pathogen resistance genes were some of the topmost significantly differentially expressed orthologous genes upon insect herbivory, and furthermore, genome-specific genes in ‘GREM4’ were also enriched in functions related to ‘plant-pathogen interactions’, while ‘PN40024’ genome-specific genes were not.

While genes implicated in pathogen resistance may appear unexpected, such genes have been reported to play roles in a variety of biotic stress and defense responses. For example, upon insect herbivory, increased expression of genes implicated in the production of disease resistance compounds, such as protease inhibitors, glucanases, chitinases, and peroxidases, have been observed in pepper, rice, and tobacco and contribute to insect resistance in other crops as well [125–133]. In a *V. riparia* hybrid grapevine, a QTL associated with phylloxera resistance was found to contain disease resistance genes, such as *Rps5* and Ca2^+^-responsive phospholipid-binding protein (*Bonzai*), supporting the idea that pathogen defense genes play a role in insect herbivory defense [57].

Overall, broadly observed up-regulation and enrichment of pathogen defense genes in ‘GREM4’ upon beetle herbivory suggests genes associated with pathogen resistance contribute to insect herbivory resistance in ‘GREM4’. It is unknown if the expression of these genes directly or indirectly contributes to the production of compounds that deter insect herbivory, help protect the plant from opportunistic pathogens that invade through the newly broken tissue, or a combination of both. Additional studies are needed to parse apart this complex interaction.

## Conclusion

In conclusion, our study determined that *V. labrusca* acc. ‘GREM4’ exhibited greater resistance to insect herbivory compared to *V. vinifera* cv. ‘PN40024’. High trichome densities found in ‘GREM4’ compared to ‘PN40024’ were shown to explain some, but not all, of the insect herbivory resistance phenotype observed of ‘GREM4’. ‘GREM4’ had higher basal expression of genes involved in defense response and secondary metabolism, likely conferring constitutive defense to insect herbivory. Under insect herbivory, genes involved in secondary metabolism, including terpene and flavonoid biosynthesis, and plant-pathogen interaction genes were enriched in ‘GREM4’, but not in ‘PN40024’, indicating the putative importance of these genes in conferring insect herbivory resistance in 'GREM4'. In ‘GREM4’ and ‘PN40024’, a comparable, but small, number of paralogous and genome-specific genes were implicated in insect herbivory defense responses, underscoring their significance. Differential expression of orthologous genes is likely the major contributor to the insect herbivory resistance phenotype observed in ‘GREM4’. In summary, these results offer support for tapping into the genetic variation of wild grapevines to enhance herbivory resistance of cultivated varieties and provide candidate genes and metabolic pathways for future investigation to determine their impact on insect herbivory resistance across plant species.

### Supplementary Information

Supplementary Material 1.

Supplementary Material 2.

Supplementary Material 3.

Supplementary Material 4.

Supplementary Material 5.

Supplementary Material 6.

Supplementary Material 7.

Supplementary Material 8.

Supplementary Material 9.

## Data Availability

The transcriptomic data underlying the results of this article are available at the NCBI BioProject PRJNA1070606 (https://www.ncbi.nlm.nih.gov/bioproject/PRJNA1070606) or by request. The genome annotation may be found on GitHub at https://github.com/cdixo/Vitis-labrusca-Version-2-Genome-Annotation.git. Custom codes used for analyses and creation of the updated genome annotation are also available on GitHub at https://github.com/cdixo/Inter-species-and-Herbivory-Publication.git.

## Abbreviations

‘GREM4’: *Vitis labrusca* acc. ‘GREM4’
‘PN40024’: *Vitis vinifera* cv. ‘PN40024’
DEG: Differentially Expressed Gene
GO Term: Gene Ontology Term
KEGG Pathway: Kyoto Encyclopedia of Genes and Genomes Pathway
ORA: Over-Representation Analysis
AOF: Area of Feeding
Δlog2FoldChange: Change in log2FoldChange
ETH: Ethylene
JA: Jasmonic Acid
SA: Salicylic Acid
QTL: Quantitative Trait Loci
