# Supplementary material for: Trichomes and unique gene expression confer insect herbivory resistance in Vitis labrusca grapevines
Source: BMC Plant Biol. 2024 Jun 27;24:609. doi: 10.1186/s12870-024-05260-9 (PMC11209964; doi:10.1186/s12870-024-05260-9)
Supplement: Supplementary file 1 — Supplementary Material 1. [file 12870_2024_5260_MOESM1_ESM.docx]

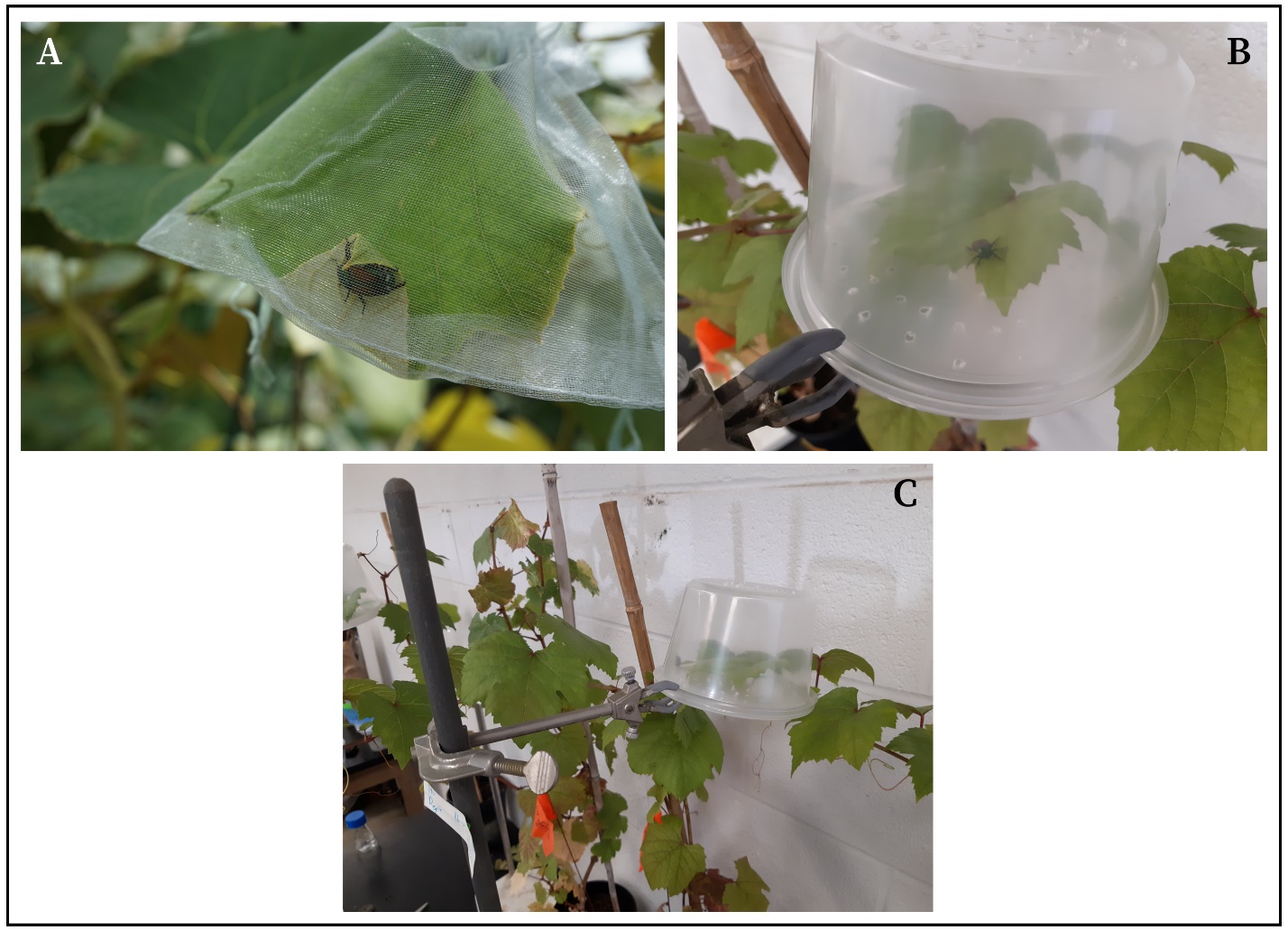


**Additional Figure 1.** Insect herbivory leaf images from the herbivory time course and trichome experiments. A. Japanese beetle in bag during an herbivory time course study run on a ‘GREM4’ grapevine leaf. B & C. Japanese beetle inside the container which was used for the herbivory under equal trichome densities and herbivory under differing trichome densities studies. Both images show ‘PN40024’ leaves from the herbivory under equal trichome densities study.


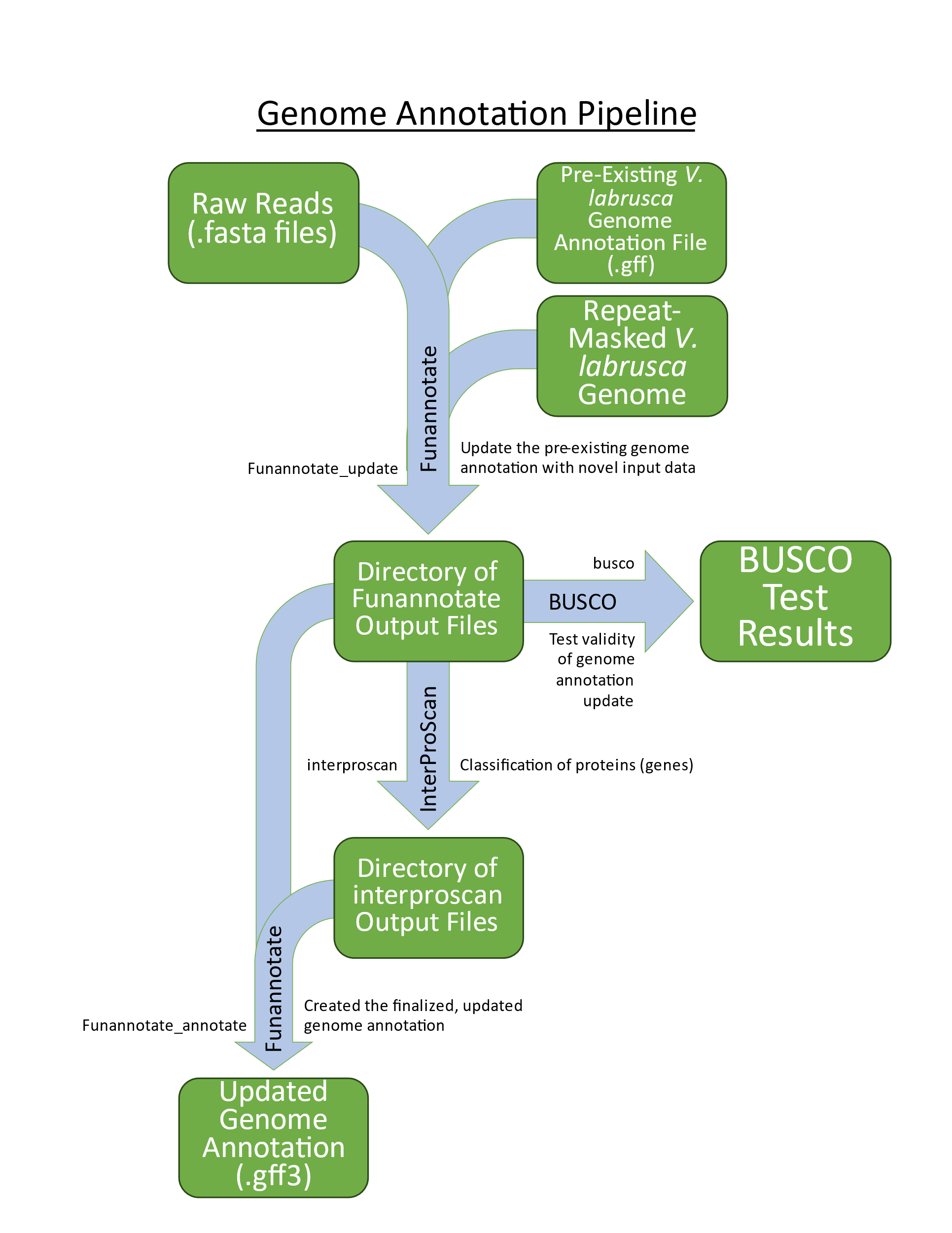


**Additional Figure 2.** Genome annotation pipeline. Bioinformatic workflow for updating the pre-existing *Vitis labrusca* acc. ‘GREM4’ gene annotation. Pipeline encompasses the addition of RNA-seq reads for additional predictive power, as well as the pre-existing gene annotation, to update the annotation of the repeat-masked V*. labrusca* ‘GREM4’ genome via Funannotate. The resulting updated genome annotation was tested with BUSCO for the presence of conserved single-copy orthologs. Green boxes represent a dataset, file, or directory while blue arrows represent a script or program being run.


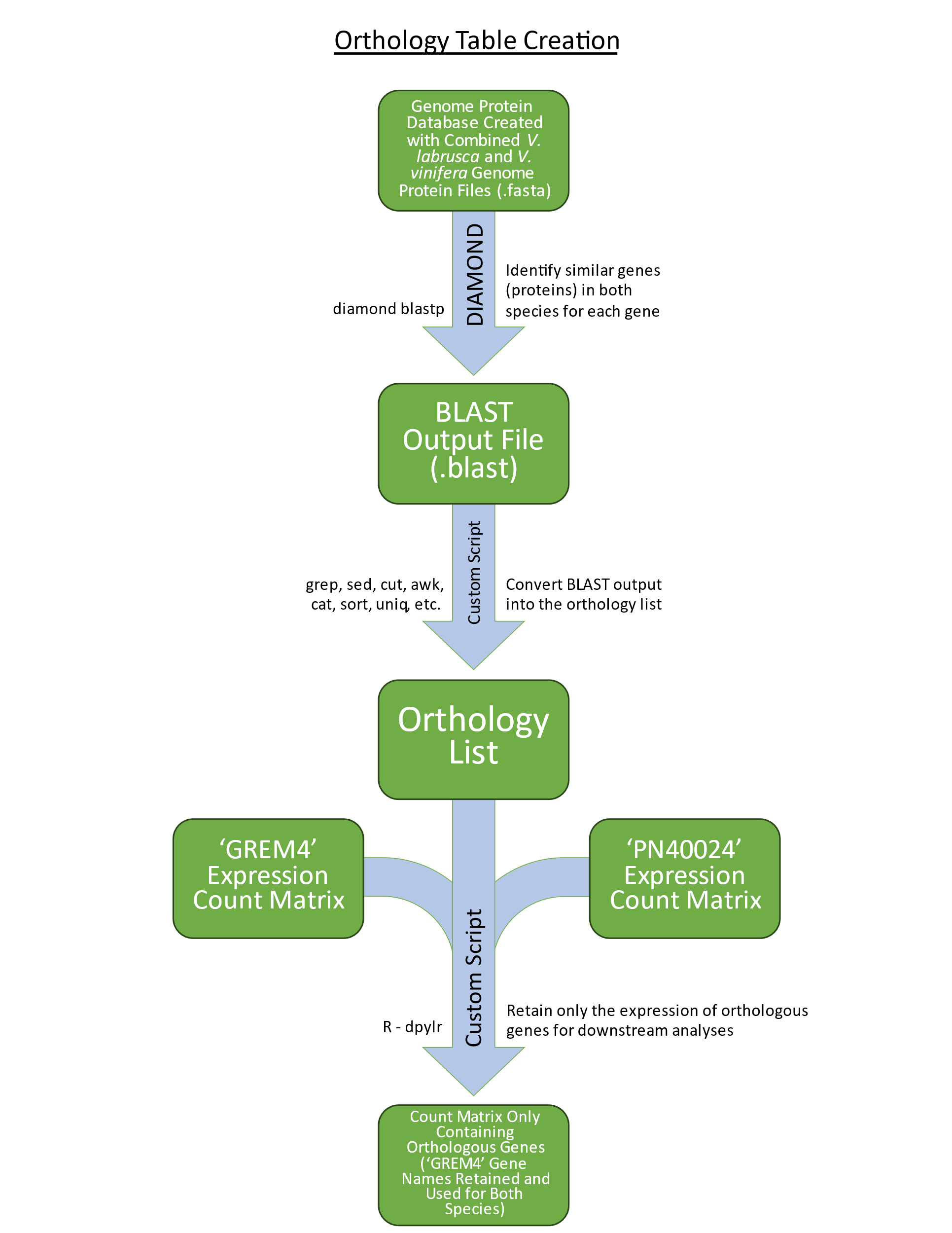


**Additional Figure 3.**  Gene orthology identification pipeline. Bioinformatic workflow for identification of orthologous genes. The resulting count matrix, which only contained orthologous genes, was used for inter-species analyses. Green boxes represent a dataset, file, or directory while blue arrows represent a script or program being run.


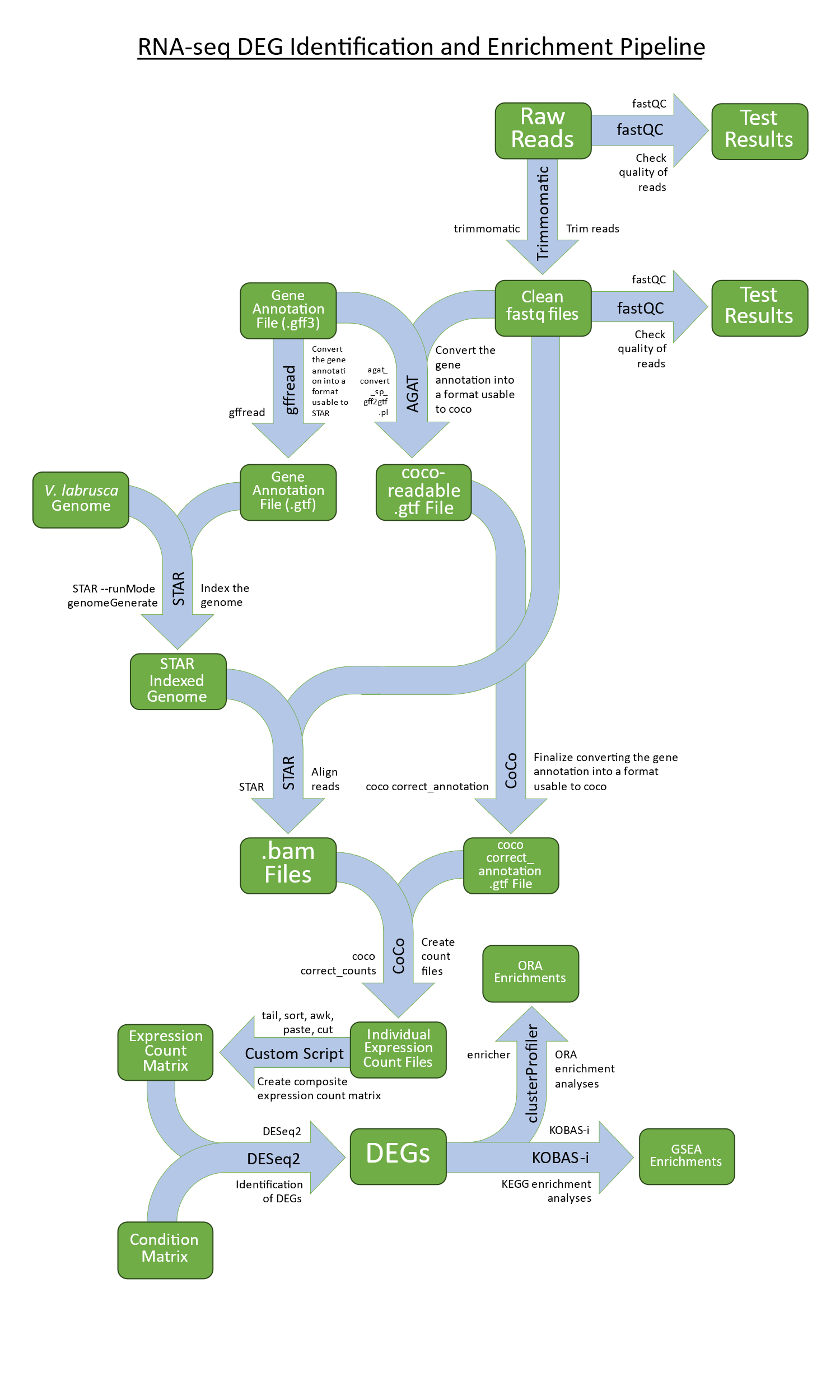


**Additional Figure 4.** RNA-seq, DEG Identification, and Enrichment Pipeline. Bioinformatic workflow for RNA-seq data analysis. Pipeline encompasses cleaning reads, quality control, read alignment, count matrix creation, DEG identification, and enrichment analysis. Green boxes represent a dataset, file, or directory while blue arrows represent a script or program being run.


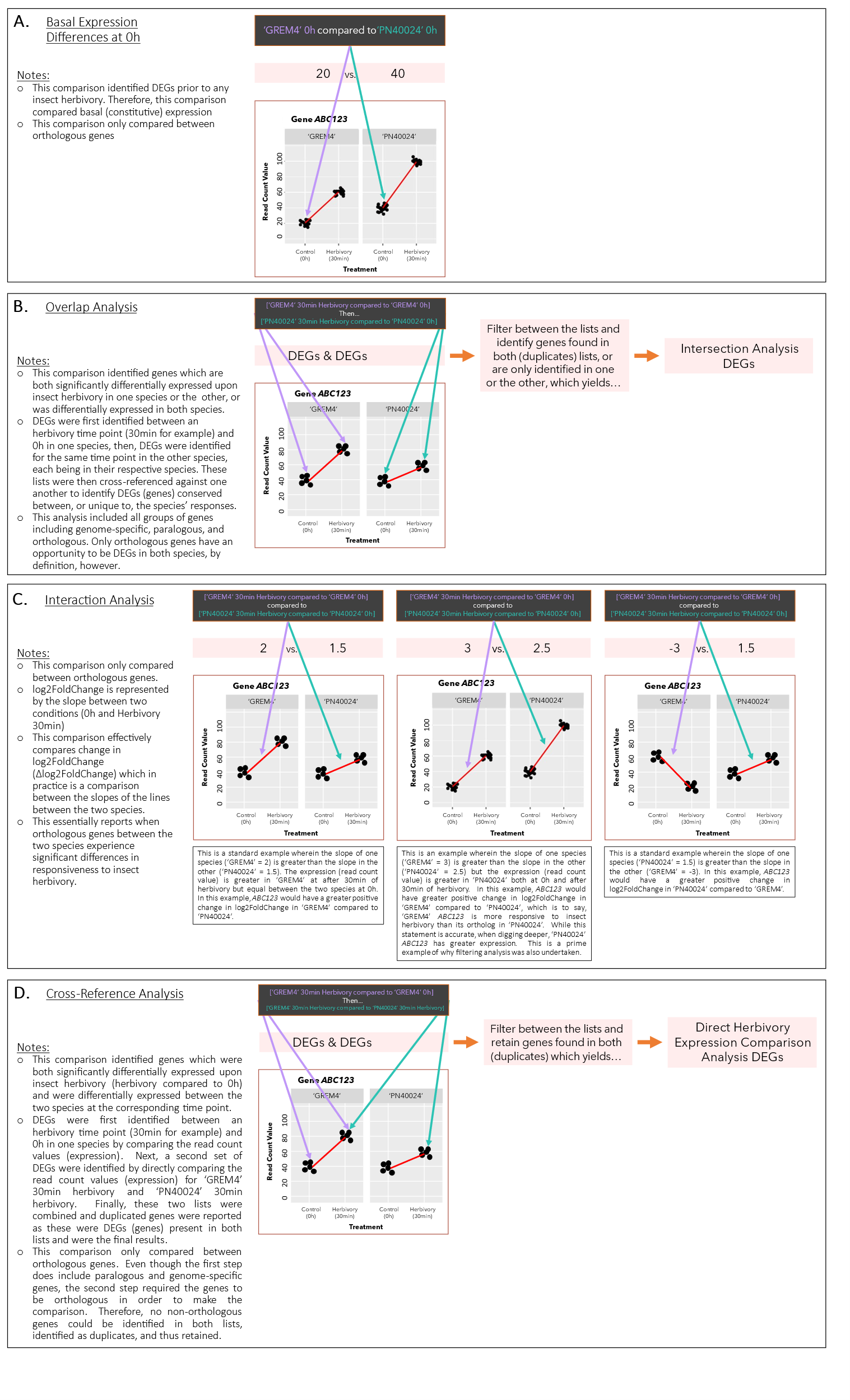


**Additional Figure 5.** Graphical depictions of inter-species comparison methods. A. Basal Expression Differences at 0h. B. Overlap Analysis. C. Interaction Analysis. D. Cross-Reference Analysis.


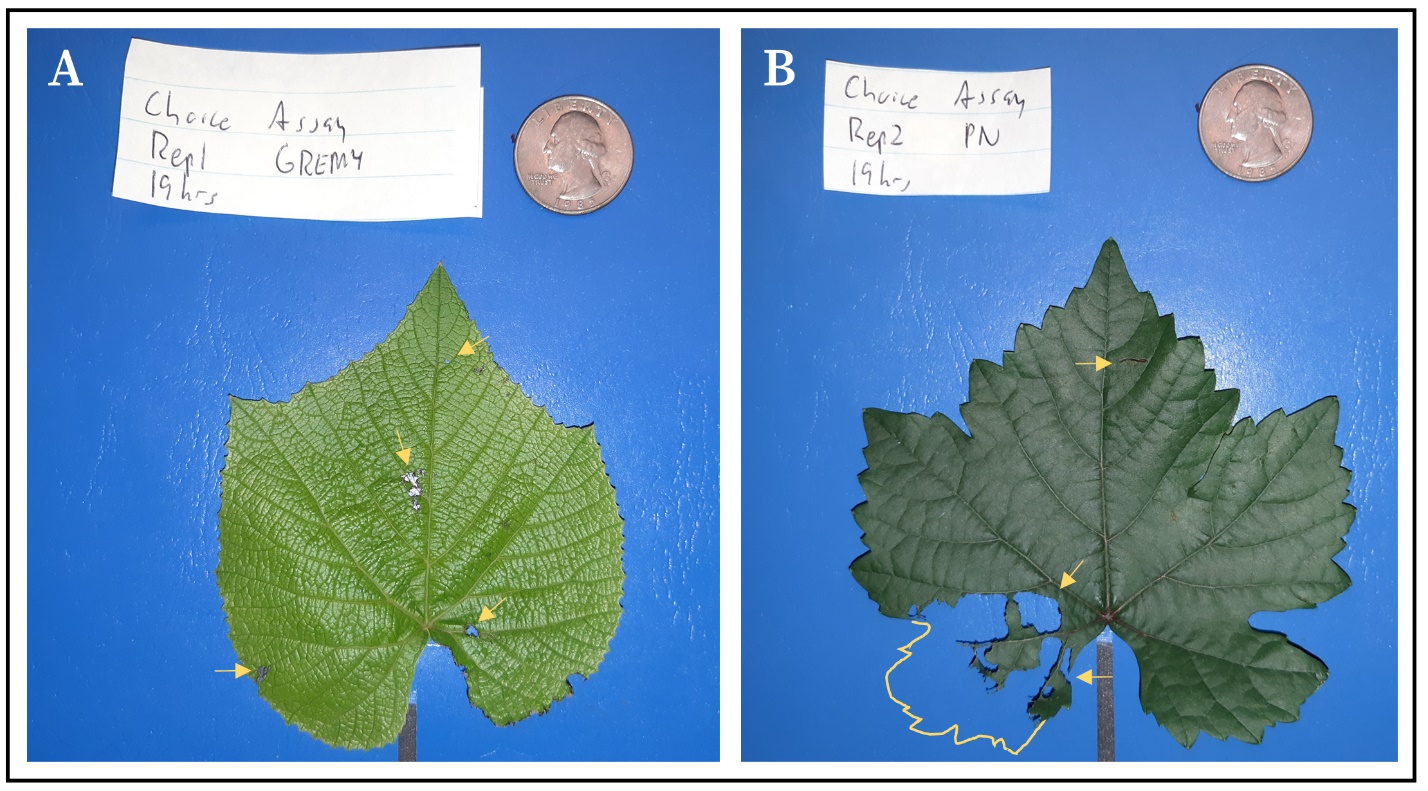


**Additional Figure 6.** Herbivory preference study feeding images. Representative images depicting damage from Japanese beetles in (A) ‘GREM4’ and (B) ‘PN40024’ leaves after 19h of feeding in the herbivory preference study. Arrows indicate locations of feeding damage while the yellow outline indicates the leaf margin before feeding. Feeding area was recorded to determine AOF.


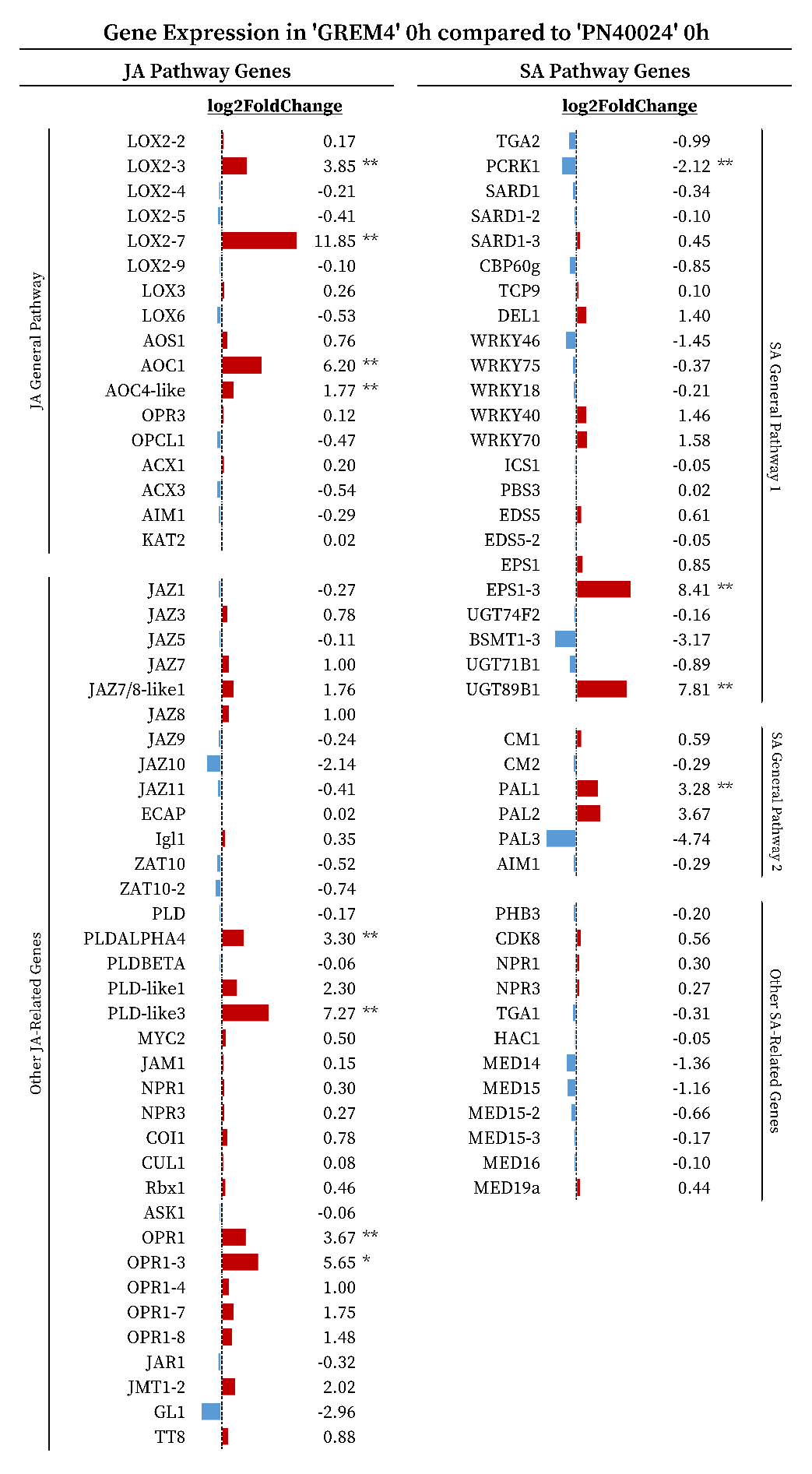


**Additional Figure 7.** JA and SA pathway gene expression in ‘GREM4’ 0h compared to ‘PN40024’ 0h. Gene expression presented via log2FoldChange. Significance presented via p-adj where * = ≤0.10 and ** = ≤0.05. Red bars indicate greater expression in ‘GREM4’ compared to ‘PN40024’ whereas blue bars indicate lower expression in ‘GREM4’ compared to ‘PN40024’. Genes are ordered by general position in each biosynthetic pathway.


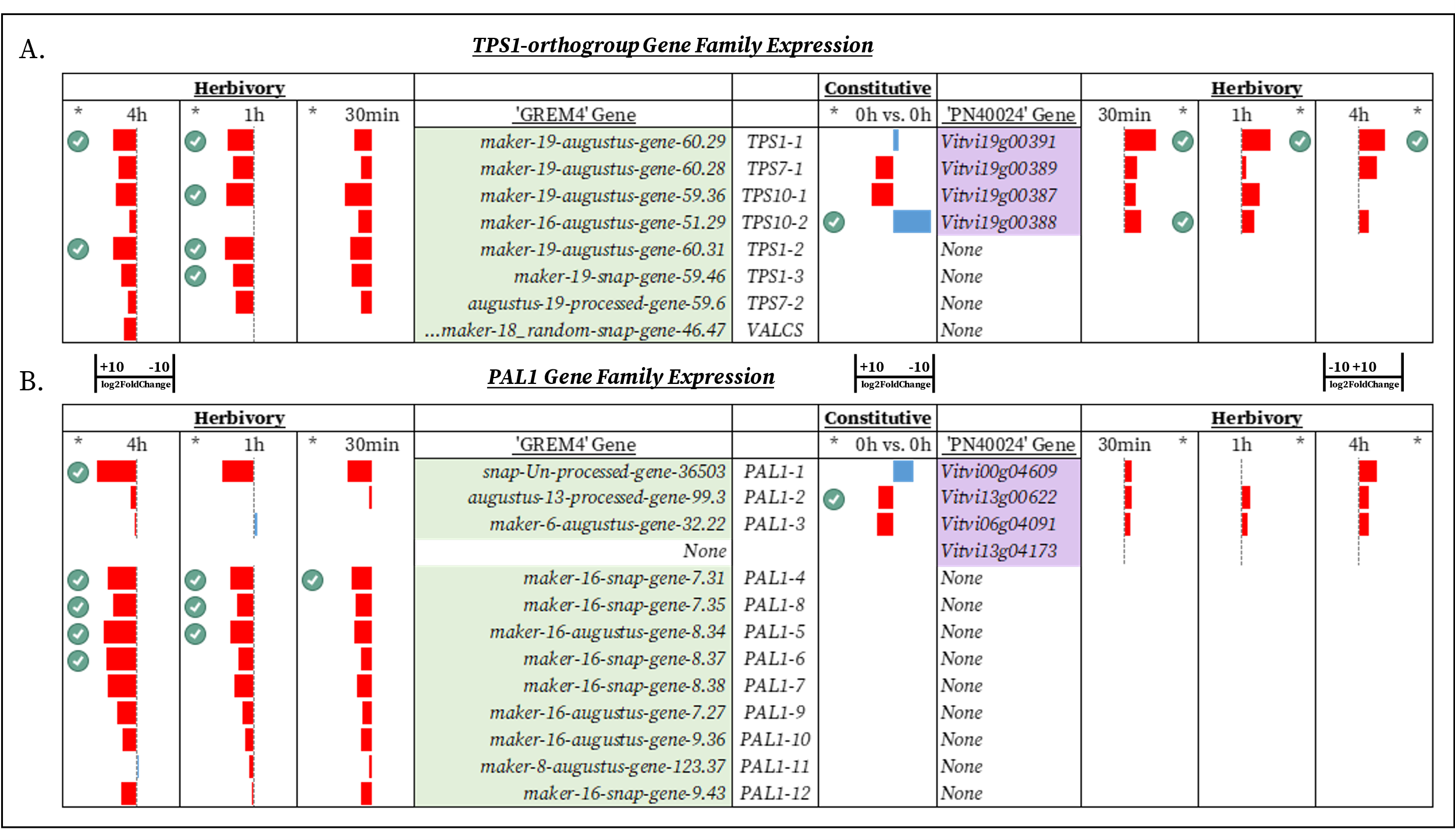


**Additional Figure 8.** Expression within expanded gene family examples. Expanded (paralogous) gene family members’ expression under insect herbivory in ‘GREM4’ and ‘PN40024’ in the *TPS1*-orthogroup (A) and *PAL1* (B) gene families. All genes in each gene family are listed along with their expression. Expression is relayed via horizontal bar graphs which illustrate expression via log2FoldChange where red is increased expression and blue is decreased. Expression is reported for both insect herbivory (insect herbivory, broken down by time point, compared to 0h) and for constitutive (basal) expression. Green check marks indicate the change in expression was significantly different for the gene family member at the respective time point where significance was determined as p-adj ≤0.05.
